# The changes in primary and secondary school teachers’ mental health level according to SCL-90 and its economic dissection—a meta-analysis with evidence from China

**DOI:** 10.3389/fpsyg.2026.1815346

**Published:** 2026-07-29

**Authors:** Yinghua Li, Jianping Xu

**Affiliations:** 1 Department of Education, Taiyuan University, Taiyuan, China; 2 Faculty of Psychology, Beijing Normal University, Beijing, China

**Keywords:** cross-temporal meta-analysis, Easterlin paradox, economic development, mental health, primary and secondary school teachers, time puzzle of mental health

## Abstract

**Objective:**

Exploring the changing trends and reasons for changes in primary and secondary school teachers’ mental health levels is of great importance, and social change, effectively represented by economic development, is a notable explanatory factor in this process. Therefore, this study focuses on the mental health levels of primary and secondary school teachers in China and analyzes the factors influencing them from an economic perspective, in order to provide a reference for the formulation of future intervention policies. Methods: A total of 153 studies published between 2000 and 2024 using SCL-90 (Symptom Checklist 90) were obtained from Web of Science, PubMed, ProQuest, Scopus, and China National Knowledge Infrastructure (CNKI). The cross-temporal meta-analysis and relevant statistical methods were used to explore the temporal and regional differences in primary and secondary school teachers’ mental health levels and their relationship with economic development.

**Results:**

From 1998 to 2021, the overall mental health levels of primary and secondary school teachers initially decreased, then slightly increased. Teachers in the eastern and northeastern regions/urban areas have better mental health levels than those in the Western China and Central China regions/rural areas, respectively. Results of the analysis of variance (ANOVA) and subgroup analysis showed that teachers’ mental health levels were related to subgroup variables for economic regions and urban/rural regions. The multiple linear regression model is not significant for the SCL-90 factors over a lag period of 0–3.

**Conclusion:**

From 1998 to 2021, the overall mental health levels of primary and secondary school teachers initially decreased, then slightly increased. There are significant regional differences in primary and secondary school teachers’ mental health levels: teachers from regions with better economic development and urban areas have higher mental health levels. From a cross-temporal perspective, the economic development level cannot well explain the changing trend of primary and secondary school teachers’ mental health levels. The “time puzzle of mental health” hypothesis proposed by this study echoes the Easterlin paradox to some extent, enriches the research in related fields, and provides a new theoretical perspective with reference value for explaining the relationship between economic development and primary and secondary school teachers’ mental health levels.

## 1 Introduction

China is actively promoting the construction of an education powerhouse. A high-quality teacher team is a strategic support for the construction of an education powerhouse (Liu et al., 2023), and teachers’ good mental health is an important foundation for building a high-quality teacher team (Huang et al., 2024). The psychological state of primary and secondary school teachers is not only the cornerstone of their career development and quality of life (Harding et al., 2019) but also has a profound impact on students’ mental health and personality development, and is an important component of the education ecosystem. Therefore, it is particularly important to understand the changing trends and factors influencing the mental health of primary and secondary school teachers.

Although some previous studies have focused on the mental health of primary and secondary school teachers, there are still shortcomings in the existing studies. First, influenced by factors such as sample size, sampling region, detection tool, and normative reference, there are differences or even contradictions among existing original studies (Yu et al., 2015; Zhang et al., 2013), which influence the public’s overall cognition of this topic. Second, for a large part of meta-analysis, the relatively short or dated sampling range make it difficult to comprehensively reflect the level and changing trend of primary and secondary school teachers’ mental health (Wang et al., 2013: 1979 ~ 2012; Wang et al., 2021: 2010 ~ 2020; Wu, 2014: 2000 ~ 2013; Xiao and Wu, 2018: 1991 ~ 2014; Zhang, 2010: 2000 ~ 2010; Zhao and Yang, 2015:1991 ~ 2010). Third, the great social changes in China in recent decades have had a significant impact on all aspects of society, including people’s mental status. However, only a few studies focus on analysis from the perspective of social change, which is not conducive to objectively and truthfully grasping the macro-level influencing factors on primary and secondary school teachers’ mental health.

To explore the cohort effect and related social change factors in the changing trend of research conclusions on the same topic, cross-temporal meta-analysis is usually used. The trend in mental health levels can be assessed by examining scores on mental factors and their changes over time (Twenge et al., 2012; Twenge et al., 2008; Xin and Chi, 2008). As a temporal symbol of social change, the epoch is closely related to factors such as the economy, politics, culture, and ecology, among which the economy is an explicit and effective indicator of social change. Thus, when exploring the changing trend and reasons for the mental health levels of primary and secondary school teachers, the level of economic development is an indispensable explanatory factor (Fan et al., 2016; Yang and You, 2017). In recent decades, China has attracted worldwide attention with its policies of reform and opening up and its rapid development. Therefore, it is notable to take China as a representative sample to discuss the relationship between changes in primary and secondary school teachers’ mental health levels and economic development.

One of the most intuitive manifestations of economic development from an individual’s perspective is a change in income level. As an effective indicator of the level of economic development of a country or region, per capita disposable income is commonly used to measure people’s living standards. Compared to “teachers’ salaries” and “educational fund,” “per capita disposable income” is a more suitable factor for measuring the level of economic development, as it is a macro indicator and data are easy to obtain while avoiding potential privacy issues. The growth rate of per capita disposable income can serve as auxiliary data for interpreting its trend. These two factors are static and dynamic indicators, respectively, for measuring the aggregate amount and the level of change. Engel’s coefficient, the share of food consumption, can, to some extent, be regarded as an indicator of income effectiveness and is often used as a measure of residents’ living standards. The interannual variation of these data focuses on changes in residents’ living standards over time, while comparisons based on the “four major economic regions (one of the commonly used and official space divisions for discussing China’s economic development)” and “urban and rural” can effectively reflect spatial differences. Given certain panel data characteristics, the aforementioned data can reflect the changes in economic development levels across time and space dimensions and serve as ideal factors for exploring the relationship between mental health and social change.

Based on the above discussion, this study used cross-temporal meta-analysis to explore changes in the mental health levels of primary and secondary school teachers in China over the past 20 years. It used a basic meta-analysis and lag analysis to examine the links between social changes and the mental health levels of primary and secondary school teachers. The per capita disposable income, the growth rate of per capita disposable income, Engel’s coefficient, and economic regionalization were introduced into the analysis as explanatory variables. The study can, to a certain extent, provide suggestions for policy and specific intervention strategies to ensure the healthy and professional development of primary and secondary school teachers, as well as teachers at other stages, and provide a theoretical reference for the continuous optimization of the education ecosystem. The discussion of the study may provide some positive insights for the teachers’ professional health development and the continuous optimization of the education ecosystem in some developing countries with similar situations.

## 2 Methods

### 2.1 Literature searching strategy

The Symptom Checklist-90 (SCL-90), a scale specifically designed to measure the overall state of mental health, was developed by Derogatis et al. in the 1970s. Since its introduction to China in the 1980s, it has gradually become the most widely used tool for investigating the psychological status of Chinese adult groups, and it is also the most widely used research tool in the existing literature on the mental health of teachers in China. Thus, in cross-temporal meta-analysis, the large sample size of the SCL-90 can help draw more accurate and ecologically valid conclusions. SCL-90 contains 10 factors (somatization, obsessive-compulsive, interpersonal sensitivity, depression, anxiety, hostility, phobic anxiety, paranoid ideation, psychoticism, and other), and measures the mental health level of the subjects over a period of time. Generally, the scale uses a 1–5 grading system, indicating five symptom states from “None” to “serious.” This study selected studies that used the Chinese version of the SCL-90 (Wang, 1984) to measure the mental health of primary and secondary school teachers as participants, and used the total score and the average score of each factor as indices for further analysis.

The search covered five databases (Web of Science, PubMed, ProQuest, Scopus, and China National Knowledge Infrastructure [CNKI]) with the search period ranging from 1 January 2000 to 31 December 2024. The eligible types were included: journal articles, conference articles, doctoral and master’s theses, and research reports. Boolean operators were used to combine the different sets of keywords. According to the criteria, documents that should be added into the dataset were those in which the title, key words or abstract contained “teacher” OR “primary school teacher” OR “secondary school teacher” OR “middle school teacher” OR “primary and secondary school teacher” OR “high school teacher” OR “senior school teacher” AND “SCL-90” OR “symptom checklist 90” OR “symptom checklist” AND “mental health,” “Chin *” was added to all English database searches, align with this study’s research focus on primary and secondary school teachers in China.

### 2.2 Screening criteria

The screening process was guided by the Preferred Reporting Items for Systematic reviews and Meta-Analyses (PRISMA) statement (Page et al., 2021). The criteria of article selection were as follows: (1) the object should be primary and secondary school teachers in China (excluding vocational school teachers, teachers from Hong Kong and Macao regions, and mixed subject teachers); (2) the type should be original research (excluding review research); (3) the tool used should be Symptom Checklist-90 (SCL-90); (4) the study should include clear quantitative analysis (generally including sample size, mean, standard deviation, etc.) and provide detailed results of SCL-90. All articles that meet the criteria above were included in the initial dataset of this study.

Studies with special samples (e.g., teachers in earthquake areas, school leaders, librarians, and care service teachers for rural left-behind children) were excluded, as these research subjects may have unique mental health levels that differ from those of the general primary and secondary school teachers and may need analysis separately. Review or analysis articles and articles with biased data (e.g., total sample and sub-sample size were seriously mismatched, mean or standard deviation was missing) were excluded. For articles published by the same author repeatedly, the earlier one was kept.

Studies with small sample sizes usually require additional attention in meta-analysis. As small samples are more likely to exhibit sampling errors, the accuracy of effect size estimates and statistical power may be affected. However, although removing studies with small sample sizes can, to some extent, make the meta-analysis reliable, the exclusion of studies may introduce bias and affect the process and the conclusion of the publication bias test. In the literature screening process for this study, only a small number of studies with small sample sizes were identified after the previous steps. Thus, these studies were added to the dataset after evaluation.

To ensure the accuracy and comprehensiveness of the review, after removing duplicate literature, the two researchers independently screened the initial dataset according to the inclusion and exclusion criteria. Preliminary screening was conducted using titles and abstracts to identify potentially relevant studies for subsequent full-text review. In the process of full-text review, by screening the reference lists of eligible studies selected in the first step, literature containing relevant data were added to the initial dataset after manual identification. After all the literature were screened and coded by researchers, consensus should be reached before a decision is made for each inconsistent screening result. For literature for which consensus could not be reached, a third researcher was temporarily brought in to make the final decision.

### 2.3 Literature coding

The studies included in this meta-analysis were coded for multiple characteristics, including publication year, sample size, publication type, economic region, and urban/rural area. In this review, the coding scheme contained two categories: descriptive information and variable information. For descriptive information, the publication year was used to correct the data collection date, and information such as article title and author(s) was recorded during the data processing and were not included in the analysis. For variable information, publication type was coded as core journal article, non-core journal article, and dissertation; economic region was coded as Eastern China, Central China, Western China, and Northeastern China. In coding economic regions and urban/rural areas, for literature that contained mixed samples or did not provide information for classification, “mixed” and “no information” were used. As the objective of this study did not include detailed comparison and analysis of teachers across different school levels, “school level” was not included in the literature coding process. For studies that included two or more independent samples that met the criteria, the samples were coded separately. Two researchers independently extracted information and completed the coding process. In cases of disagreement or inconsistency, a third researcher was involved temporarily to reach consensus.

### 2.4 Data preprocessing

The majority of the collected literature establishes the analysis based on the total samples. For literature used classified samples (e.g., gender, nationality, educational background, title, etc.), to improve utilization of literature, the SCL-90 data was added to the dataset after weighting by using Equation 1 and Equation 2:


$$\bar{x}=\frac{\sum x_{i}n_{i}}{\sum n_{i}}$$ (1)

$$S_{T}=\sqrt{\frac{\sum n_{i}s_{i}^{2}+\sum n_{i}{\left(x_{i}-\bar{x}\right)}^{2}}{\sum n_{i}}}$$ (2)

The majority of the selected literature used a 1–5-point scoring system to measure SCL-90 results. For the literature used 0–4-point scoring system, 1 was added to each factor to ensure data consistency. For literature that did not clearly mention A scoring system, checked the scoring method by referring to their SCL-90 judgment criteria, and then completed data alignment.

There are four economic regions in China’s economic statistics: Eastern China, Western China, Central China, and Northeastern China. Literature without sample location information were excluded when conducting regional comparisons and analyses. For literature that used mixed samples, those containing classified samples and corresponding SCL-90 scores were added to the dataset for comparison after sample splitting.

For literature in which the author clearly stated the survey time, the date mentioned in the literature was used directly. For literature without a clear date of data collection, the “publication date minus 2” principle was used to standardize the data collection date. If the data collection process was relatively long and the start and end times were not in the same year, the finish time was used.

The economic data were obtained from the China Statistical Yearbook, the most authoritative and comprehensive compilation of China’s annual statistics published by the National Bureau of Statistics. It systematically collects economic and social data for the whole nation, as well as for each province, autonomous region, and municipality directly under the central government, along with the main indicators from previous years. It is an informative annual publication that comprehensively reflects the economic and social development of the People’s Republic of China. The economic data required in this study (per capita disposable income, growth rate of per capita disposable income, Engel’s coefficient) can be found in the Price and People’s Livelihoods sections of the yearbook. The year in the yearbook name usually represents the year of publication, and its content is data from the previous year. Thus, the yearbooks needed for this study were from 1999 to 2023. The national statistical data in the yearbook, unless otherwise specified, do not include data from the Hong Kong Special Administrative Region, Macao Special Administrative Region, and Taiwan Province. As this study focused on primary and secondary school teachers in China, this feature of the yearbook’s data did not require additional processing.

### 2.5 Quality assessment of studies

Heterogeneity was evaluated using Cochran’s Q and the I^2^ index, with I^2^ < 25, 25% ≤ I^2^ ≤ 50, 50% ≤ I^2^ ≤ 75%, and I^2^ ≥ 75% indicating low, moderate, high, and very high heterogeneity, respectively. The 2006 norm (Tong, 2010) was used to evaluate the teachers’ relative mental health levels. As the selected studies used the same scale and the scoring rules have been standardized, mean difference (MD) and inverse-variance weighting methods were used to calculate the effect size and study weight.

The JBI (Joanna Briggs Institute) Assessment of Risk of Bias for Analytical Cross-sectional Studies was introduced to assess the quality of the selected studies (Barker et al., 2026). The JBI assessment tool contains 8 items for, respectively, judging the bias related to internal validity, statistical conclusion validity and comprehensiveness of reporting: clear criteria for sample inclusion (I1), objective, standard criteria used for measurement (I2), valid and reliable measurement of exposure (I3), valid and reliable measurement of outcomes (I4), identification of confounding factors(I5), strategies to deal with confounding factors (I6), appropriate statistical analysis (I7), detailed description of study subjects and the setting (I8). For items in the JBI tool, “Yes,” “No,” “Unclear,” and “Not applicable” are used for judgment. According to the principles of the JBI tool, studies were classified into three quality levels based on score: high quality (≥6 points), moderate quality (4–5 points), and low quality (≤3 points). Two researchers independently evaluated the eight items included in the JBI tool. In cases of disagreement or inconsistency, a third researcher was involved temporarily to reach consensus.

To reduce the impact of potential bias and improve the reliability and validity of the research results, a publication bias test was introduced. In this study, publication bias was first evaluated using funnel plots and then rigorously tested using the Fail-Safe Number (N_fs_) Egger’s regression (Borenstein et al., 2013). The Fail-Safe Number (N_fs_) indicates how many negative studies (i.e., zero or near-zero effect sizes) would need to be supplemented, without changing the existing meta-analysis conclusions, to eliminate the significance of the overall effect size. Fail-Safe Number, to some extent, reflects the possibility of publication bias. Generally, a larger Fail-Safe Number indicates a lower likelihood of publication bias. The commonly used minimum acceptable value for the safety factor is 5 k + 10. The core concept of Egger’s regression is that if there is no significant publication bias, the effect sizes of the studies should be independent of accuracy (reciprocal of the standard error), and the intercept term of the regression equation should not significantly deviate from 0. In this study, Egger’s regression was regarded as a supplement and test of the funnel plot.

### 2.6 Statistical methods

Due to significant differences in the survey time and sample source regions of the selected literature, the results of the heterogeneity test are likely to show relatively high heterogeneity. Therefore, subgroup analysis was introduced as part of the toolset to discuss changes over time and regional differences in SCL-90 scores. A comparison between the total 153 studies and the studies set for subgroup analysis was conducted to ensure the rationality and robustness of the subgroup analysis.

Analysis of variance (ANOVA) was used to test whether a categorical independent variable has a significant impact on a continuous dependent variable. It was introduced in this study to analyze the regional differences (differences among four economic regions and between urban and rural areas) in SCL-90 scores, together with subgroup analysis. Normality and homogeneity of variance tests were conducted before the data were used for one-way analysis of variance.

Linear regression was used to analyze the quantitative relationship between the dependent variable and one or multiple independent variables. Multiple linear regression was used in this study to examine the temporal relationship between economic development (per capita disposable income, per capita disposable income growth rate, and Engel’s coefficient) and the mental health levels of primary and secondary school teachers. In Equation 3, $MS_{i}$ , $I_{i}$ , $IR_{i}$ , $\;\text{and}\;EC_{i}$ are value of a SCL-90 factor, per capita disposable income and its growth rate and Engel’s Coefficient in the *i*th year, respectively; $\alpha_{i}$ and $\varepsilon_{i}\;$ are the constant term and random error term, respectively. Lag analysis is one of the basic logics of cross-temporal meta-analysis. With reference to previous research, a time lag of 0–3 years was incorporated into the calculation of the multiple linear regression model (Xin and Chi, 2008). The variance inflation factor and the Durbin-Watson test were used to assess the existence of collinearity and temporal autocorrelation, respectively.


$$MS_{i}=\beta_{1}I_{i}+\beta_{2}IR_{i}+\beta_{3}EC_{i}+\alpha_{i}+\varepsilon_{i}$$ (3)

## 3 Result

### 3.1 Studies selection process and basic characteristics of included studies

**Figure 1:**
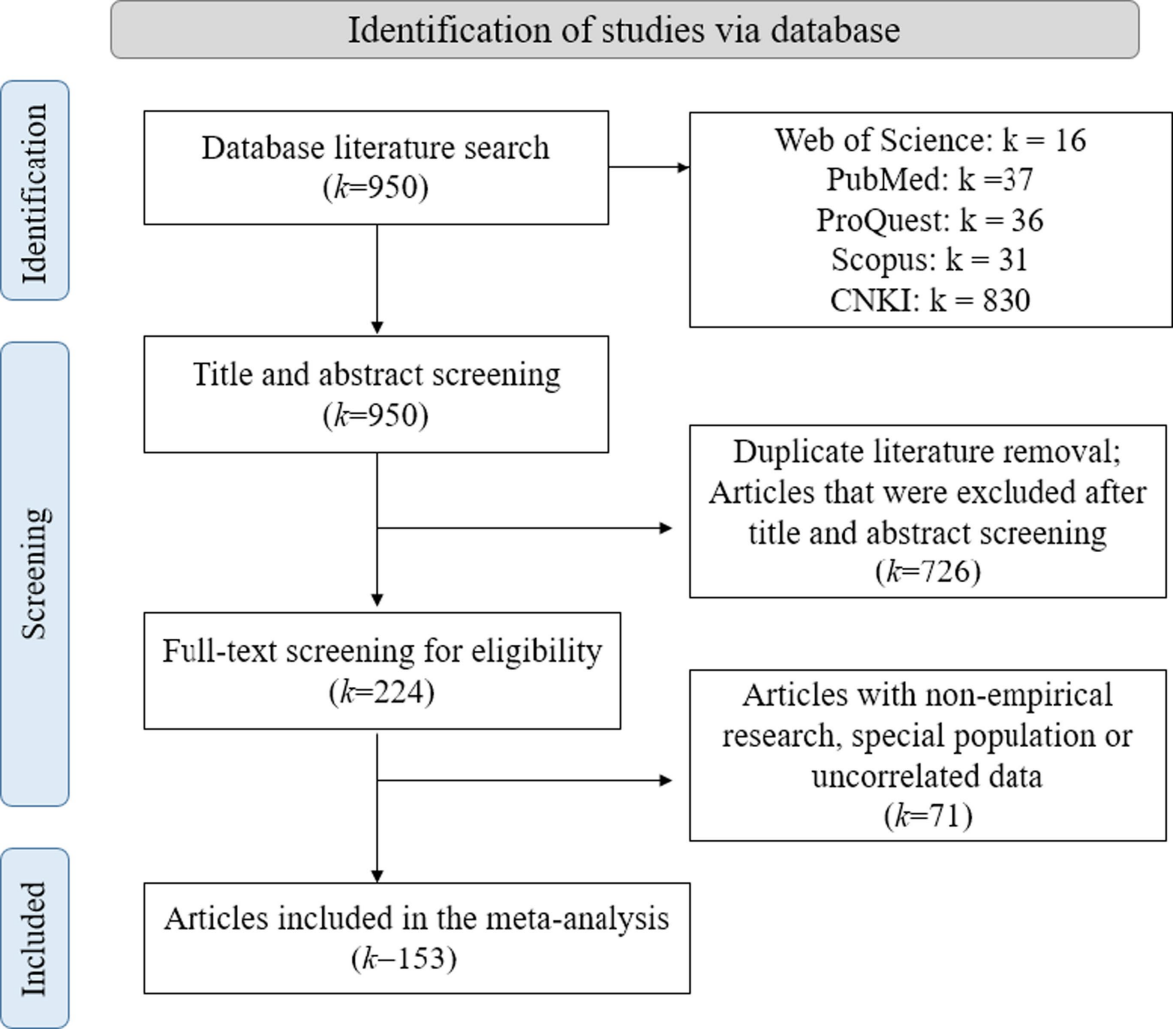
Flow chart of the literature screening process.

**Figure 2:**
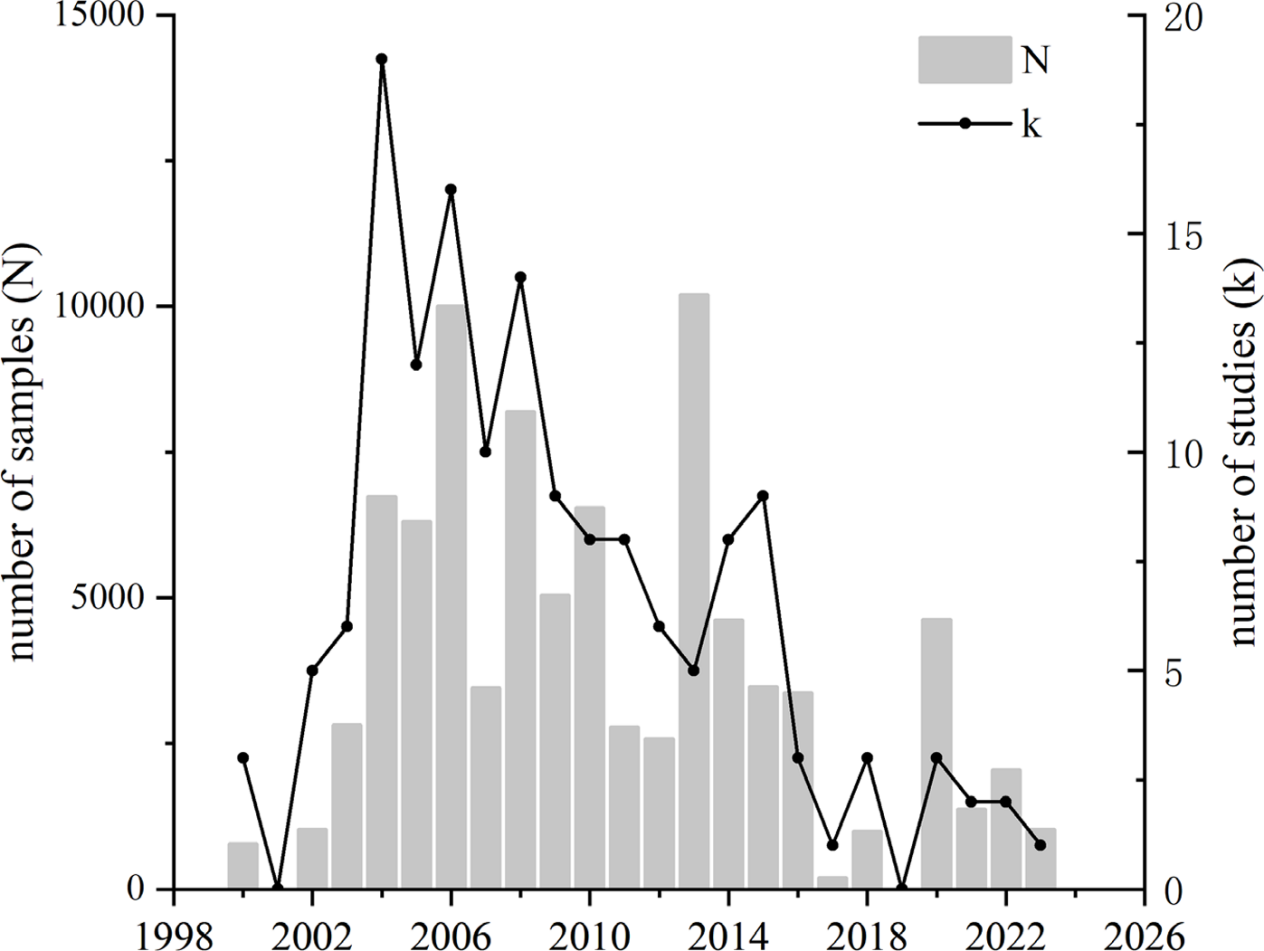
Publication year and sample distribution of selected literature.

According to the searching strategy and inclusion–exclusion criteria in the PRISMA diagram (see Figure 1), a total of 153 articles were included in the database for further analysis after the searching and screening process, including 129 journal articles and 24 dissertations, with a total effective sample size of 8,8,170 and a publication year span from 2000 to 2023 (research year span from 1998 to 2021) (see Figure 2).

In line with the study’s objectives, the basic characteristics of the included literature were extracted and summarized. All data were coded by sample size, research year, publication types, economic regions, and urban/rural areas, and the number of literature and sample sizes for each coding were calculated (see Table 1; Supplementary file S1).

**Table 1:** Literature coding (k = 153, *N* = 88170).

Variables	Code	Number of datasets (k)	Number of samples (*N*)
Publication type	1 = Non-core journals	73	45,169
	2 = Core journals	57	32,815
	3 = Dissertations	23	10,186
Economic regions	1 = Eastern China	47	21,150
	2 = Central China	36	17,727
	3 = Western China	51	32,505
	4 = Northeastern China	12	5,909
	0 = Mixed/no information	7	10,879
Urban/rural	1 = Urban	59	37,170
	2 = Rural	40	21,400
	3 = Mixed	29	14,734
	0 = No information	25	14,866

### 3.2 Quality assessment and publication bias test

Two independent reviewers assessed each study with high inter-rater reliability (agreement in the literature: 140/153, overall agreement rate: 91.5%). Inconsistency was resolved through discussion and consultation with a temporary third reviewer. According to the JBI tool evaluation results, among the 153 studies, 126 were classified as high quality (6–8 points), and 27 as moderate quality (4–5 points) (see Supplementary file S3). The majority of the selected studies’ quality was “high.” Some studies are judged to have concerns regarding the description of subjects and identification and adjustment of confounding factors; however, there was no conclusive evidence to indicate that these studies have significant flaws. In conclusion, the selected studies were found to have a low risk of bias.

**Figure 3:**
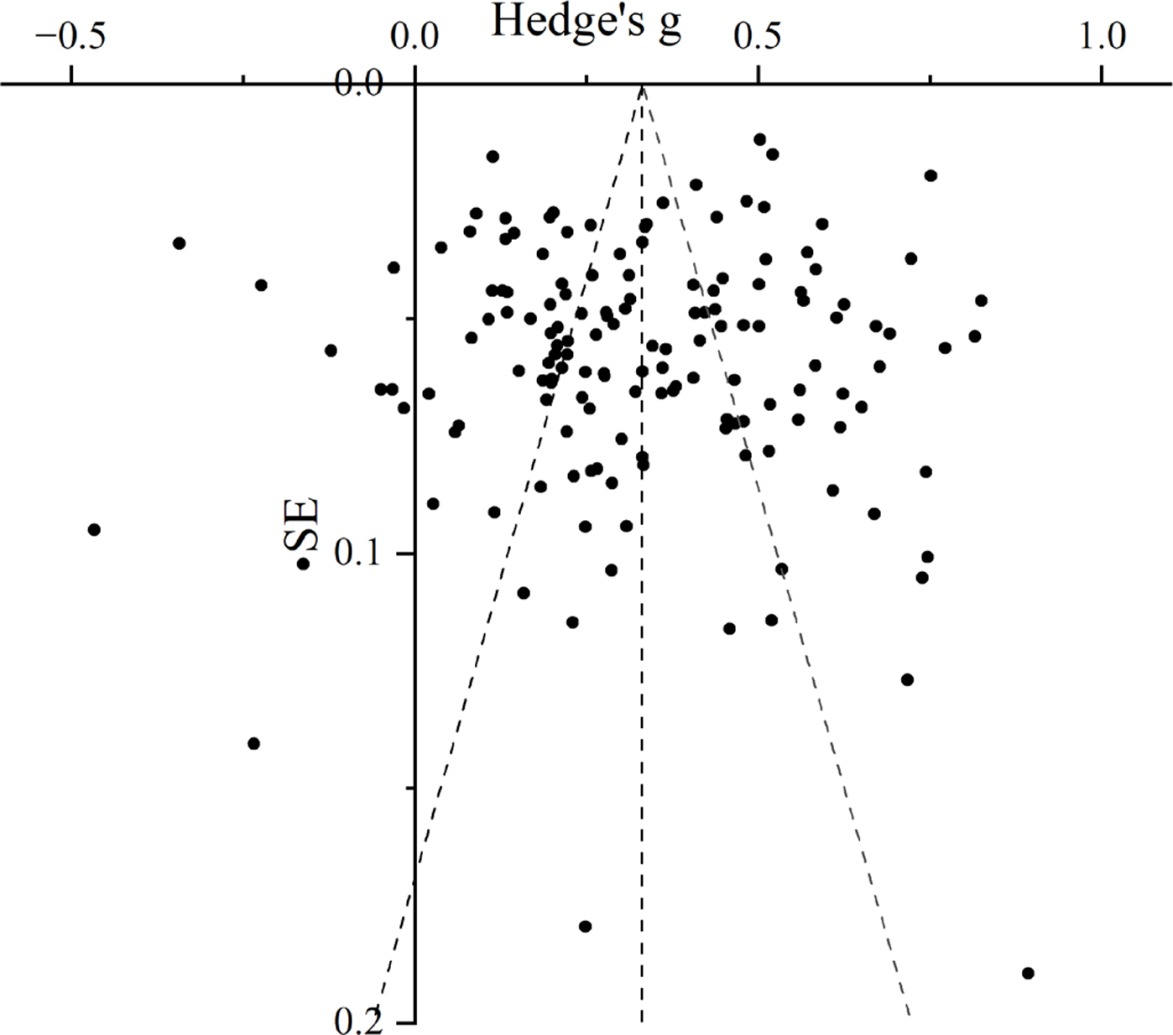
Funnel plot of meta-analysis of published studies.

The funnel plots showed that effect sizes of most selected studies were concentrated in the upper-middle region and were generally symmetrically distributed, and only a few studies were scattered at the bottom or significantly asymmetrical (see Figure 3), indicating that the possible unpublished or missing studies have a limited impact on the overall effect size. For the evaluation of the SCL-90 score, the N_fs_ value was 3,593, which was much greater than the critical value of 5 k + 10 in this study (775), suggesting that the number of unpublished and missing studies may not be large enough to significantly affect the current conclusions. The *p*-value of Egger’s test was greater than 0.05, indicating that the intercept was not significantly deviated from 0 (see Table 2). Thus, there was only a low likelihood of publication bias, and the overall findings were relatively stable and unlikely to be overturned by unpublished or missing studies.

**Table 2:** Results of Egger’s test and fail-safe number.

Intercept	95% CI		*p*	N_fs_
	Lower limit	Upper limit		
1.473	−0.609	3.556	0.164	3,593

$N_{\mathrm{fs}}={\frac{\sum Z_{i}}{1.64}}^{2}-k,(p=0.05)$ ; MD, mean difference; CI, confidence interval.

### 3.3 The overall status and changing trend of primary and secondary school teachers’ mental health level

**Figure 4:**
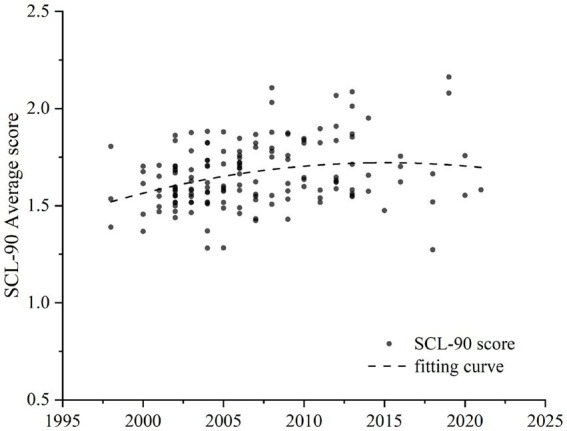
The SCL-90 scores of primary and secondary school teachers from 1998 to 2021.

To visually describe the overall changing trend of Chinese primary and secondary school teachers’ mental health level, a scatter plot was drawn with the year as the horizontal axis and the SCL-90 total score as the vertical axis (see Figure 4). Based on the selected studies’ results and the changing trend of the fitting curve, and to some extent taking into account the impact of extreme values, it can be concluded that from 1998 to 2021, the SCL-90 average score decreased first and then slightly increased, showing an overall slow downward trend of primary and secondary s chool teachers’ mental health level. The peak of the SCL-90 average score appeared around 2012 and 2013.

The effect size *d* was used to assess the change in the mean values of various SCL-90 factors (see Table 3). From 1998 to 2012, the range of the mean values of various factors was 0.087–0.360, with an average increase of 0.136–0.540 standard deviations (*d*), or a growth of 0.5–6.8% (*r^2^*). Based on the differentiation of effect sizes (threshold values of 0.2, 0.5, and 0.8 for small, medium, and large effects, respectively) (Cohen, 1977), it could be seen that for the changes in various SCL-90 factors between 1998 and 2012, the effect size of somatization was medium, and the effect sizes of interpersonal sensitivity, hostility, and paranoid ideation were not significant. Effect sizes of other factors were small. Among them, the changes in somatization and compulsion were more significant, with effect sizes of 0.540 and 0.351, respectively. From 2012 to 2021, the range of the mean values of various factors was from −0.068 to −0.206, indicating a slight improvement in mental health levels, with an average decrease from 0.113 to 0.351 standard deviations (*d*), or from 0.3–3% (*r^2^*). Between 2012 and 2021, the effect sizes of somatization, interpersonal sensitivity, anxiety, paranoid ideation, and psychoticism were small, and the effect sizes of other SCL-90 factors were not significant.

**Table 3:** Chronological changes of SCL-90 factors from 1998 to 2021 (*N* = 88,170).

Factors	Chronological changes					
	*M*_1_	*d_1_*	*r_1_^2^*	*M*_2_	*d_2_*	*r_2_^2^*
Somatization	0.360	0.540	0.068	−0.191	−0.282	0.019
Obsessive-compulsive	0.231	0.351	0.030	−0.094	−0.143	0.005
Interpersonal sensitivity	0.098	0.149	0.005	−0.161	−0.264	0.017
Depression	0.162	0.241	0.014	−0.114	−0.177	0.008
Anxiety	0.162	0.264	0.017	−0.121	−0.198	0.010
Hostility	0.119	0.181	0.008	−0.072	−0.113	0.003
Phobic anxiety	0.125	0.218	0.012	−0.068	−0.121	0.004
Paranoid ideation	0.087	0.136	0.005	−0.206	−0.351	0.030
Psychoticism	0.163	0.281	0.019	−0.134	−0.251	0.016

M_1_ = M_2012_ − M_1998_, M_2_ = M_2021_ − M_2012_, d_1_ = M_1_/SD_1998 ~ 2012_, d_2_ = M_2_/SD_2012 ~ 2021_, $r^{2}={\left(\frac{d}{\sqrt{d^{2}+4}}\right)}^{2}$ .

Substantial heterogeneity was observed across the selected studies (I^2^ = 96.96%), and a random-effects model was applied due to the high heterogeneity (see Table 4; Supplementary file S4). The effect size indicated that the mental health levels of primary and secondary school teachers were worse than the national norm (MD = 0.211, 95% CI: [0.184, 0.237]).

**Table 4:** Heterogeneity test.

MD	Q	*p*-value	95% CI	I^2^	τ^2^
0.211	4768.19	<0.001	[0.184, 0.237]	96.96%	0.026

MD, mean difference; CI, confidence interval.

### 3.4 The relationship between economic development and the mental health level of primary and secondary school teachers

**Figure 5:**
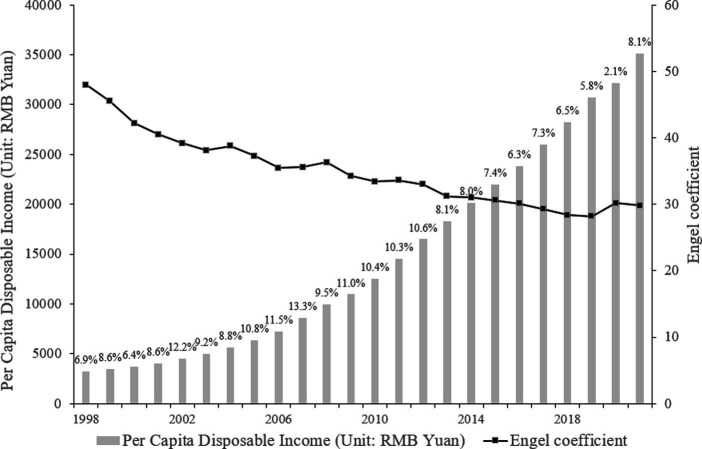
Changing trend of the selected economic factors.

To visually present the economic development status, the trends in per capita disposable income growth rate, per capita disposable income growth rate, and Engel’s coefficient were analyzed. The results showed that from 1998 to 2021, the per capita disposable income of residents continued to grow, while its growth rate fluctuated and saw an overall downward trend, indicating a slowing down in income growth (see Figure 5). Engel’s coefficient also fluctuated, but overall, it showed a downward trend, indicating that the residents’ economic level was continuously increasing.

**Figure 6:**
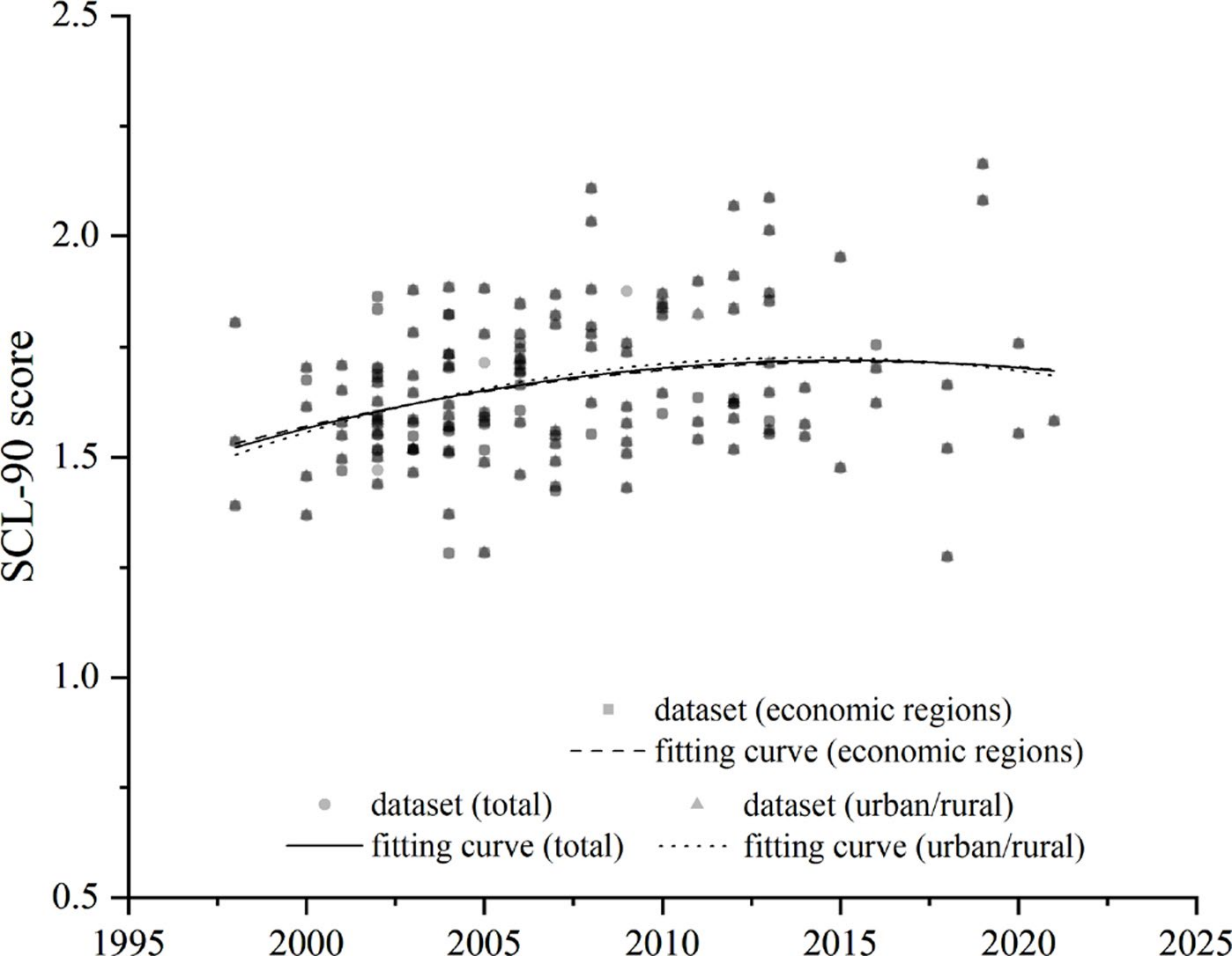
Changing trend of the subgroup datasets.

There was no significant difference between the changing trend of the subgroup datasets and the total samples (see Figure 6). The results of heterogeneity show no significant difference between datasets (see Table 5). Therefore, the comparison indicates that the subgroup dataset (samples without economic region or urban/rural area information were excluded) is sufficiently rational and robust for further subgroup analysis.

**Table 5:** Heterogeneity test for subgroups.

Dataset	k	MD	Q	*p*-value	95% CI	I^2^	τ^2^
Total	153	0.211	4,768.19	<0.001	[0.184, 0.237]	96.96%	0.026
Economic region	147	0.210	4,618.64	<0.001	[0.183, 0.237]	97.19%	0.026
Urban/rural	114	0.223	3,505.06	<0.001	[0.192, 0.255]	97.12%	0.028

k, number of studies; MD, mean difference; CI, confidence interval.

The subgroup analysis showed that the SCL-90 score of each subgroup was higher than the national norm (MD > 0), and economic regions and urban/rural areas significantly moderated the effect sizes (economic region: *p* < 0.01, urban/rural: *p* < 0.05) (see Table 6). The inter-subgroup difference was notable, indicating that there may exist a regional difference in teachers’ mental health levels. The effect size (degree of deviation from the norm) was ranked from low to high as follows: Eastern China (MD = 0.135), Northeastern China (MD = 0.178), Central China (MD = 0.241), Western China (MD = 0.264).

**Table 6:** Subgroups analysis.

Subgroup	k’	MD	SE	95% CI	I^2^	Between-group difference
Eastern China	48	0.135	0.023	[0.089, 0.180]	96.53%	Q_m_(3) = 18.17 *p* < 0.001
Western China	52	0.264	0.025	[0.215, 0.314]	97.76%	
Northeastern China	12	0.178	0.045	[0.079, 0.277]	96.54%	
Central China	36	0.241	0.023	[0.193, 0.288]	94.59%	
Urban	74	0.191	0.017	[0.158, 0.224]	95.78%	Q_m_(1) = 5.71 *p* < 0.05
Rural	55	0.256	0.021	[0.213, 0.299]	95.58%	
1998–2003	45	0.149	0.018	[0.113, 0.185]	94.38%	Q_m_(3) = 15.74 *p* < 0.001
2004–2009	64	0.211	0.021	[0.170, 0.251]	97.27%	
2010–2015	33	0.278	0.030	[0.218, 0.338]	97.90%	
2016–2021	11	0.253	0.075	[0.085, 0.421]	98.97%	

k’, number of studies for subgroup (including valid mixed samples); MD, mean difference; SE, standard error; CI, confidence interval.

The normality test showed that the data distribution did not have normality, but still presented a “bell-shaped” distribution, and the Levene test showed that the data exhibited homogeneity of variance. Therefore, analysis of variance could be used in further research (see Table 7; Supplementary file S5). The results of the F-test showed significant differences in the mean of the SCL-90 factors among primary and secondary school teachers from different economic regions (except hostility). For the analysis of urban/rural areas, the results of the F-test showed significant differences in the mean values of the SCL-90 factors (except Somatization and Hostility). The reports on regional differences across studies were generally consistent, showing that the class variables of economic regions and urban/rural areas can moderate teachers’ mental health levels. The results of ANOVA were generally consistent with subgroup analysis.

**Table 7:** Analysis of teachers’ SCL-90 factors from the four major economic regions and urban/rural areas.

Factors	Economic regions (k’ = 148)		Urban/rural areas (k’ = 129)	
	Levene	*F*	Levene	*F*
Somatization	2.167	5.261**	0.362	0.313
Obsessive-compulsive	1.057	5.941**	0.678	5.134*
Interpersonal sensitivity	1.425	3.342*	0.698	5.084*
Depression	2.346	6.142**	0.000	4.924*
Anxiety	1.523	4.519**	0.305	4.736*
Hostility	1.410	2.253	0.103	1.227
Phobic anxiety	1.175	6.770**	0.434	6.381*
Paranoid ideation	1.640	4.785**	0.768	6.608*
Psychoticism	0.230	6.444**	0.674	8.590**
Total score	1.401	6.021**	0.027	6.111*

^*^p < 0.05, ^**^p < 0.01; k’, number of studies for subgroup (including valid mixed samples).

**Figure 7:**
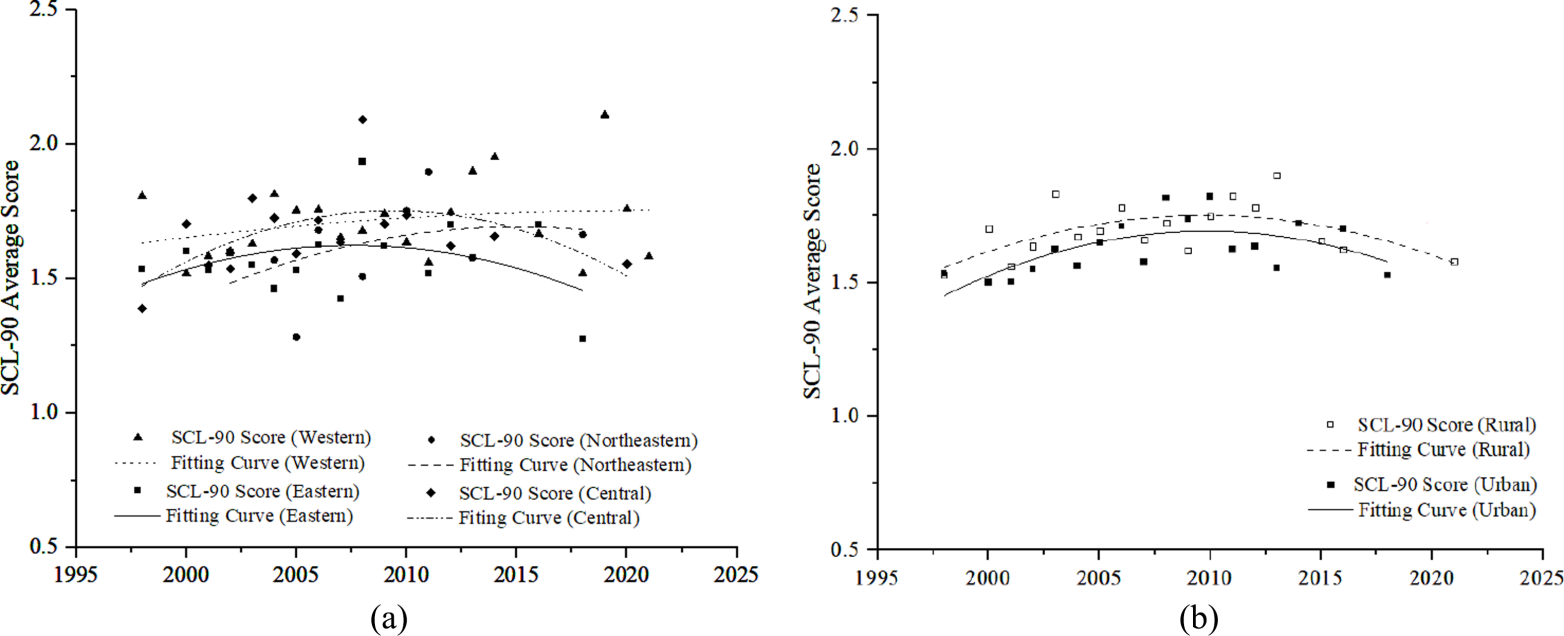
Annual SCL-90 average score. **(a)** Four major economic regions; **(b)** urban/rural areas.

From the organized annual economic region data in the scatter plot of annual SCL-90 average scores, it can be found that the SCL-90 scores of teachers in Western China and Central China regions were high. In contrast, teachers in Eastern China regions had the lowest SCL-90 scores, indicating that there existed inter-regional differences in mental health levels. In more than 79% of the economic region comparison, teachers in regions with better economic development status had relatively lower SCL-90 scores, and in more than 70% of the urban–rural comparison, the SCL-90 scores of urban teachers were lower than those of rural teachers in the same year. The changing trend of each curve was generally consistent with the overall trend of the selected data, and the fitting curves showed that there was some correlation between mental health level and regional economic development (see Figure 7).

**Figure 8:**
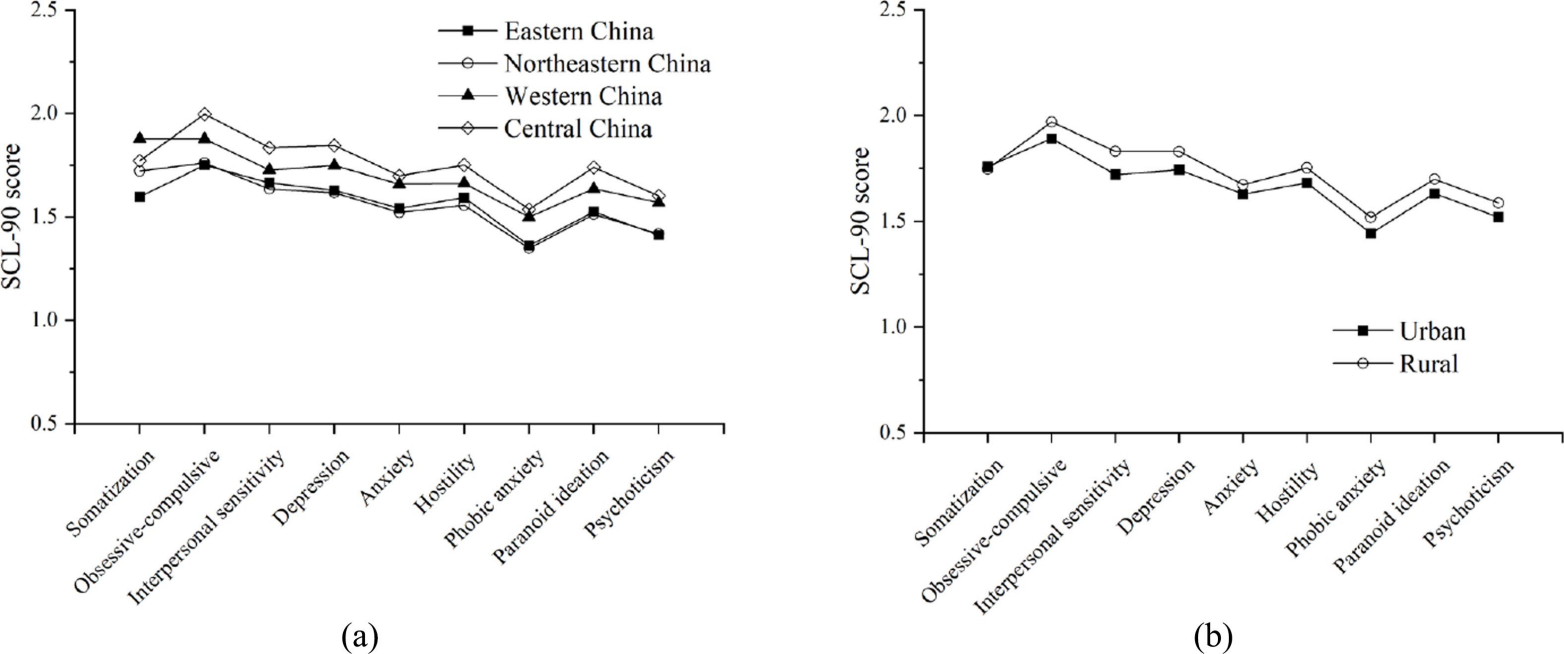
SCL-90 factors of primary and secondary school teachers. **(a)** Four major economic regions; **(b)** urban/rural areas.

By drawing a line chart to visually describe the relationship between economic development and the mental health level (see Figure 8), the results showed that the SCL-90 scores of primary and secondary school teachers across different economic regions were ranked from low to high as follows: Eastern China (*M*: 1.36–1.75), Northeastern China (*M*: 1.35–1.76), Western (*M*: 1.50–1.88), and Central (*M*: 1.54–2.00). This indicated a regional difference among the four major economic regions: teachers from regions with better economic development have higher levels of mental health. There was also a regional difference between SCL-90 scores of factors in urban and rural areas (*M*_urban_: 1.44–1.89, *M*_rural_: 1.52–1.97), indicating that the mental health level of teachers in urban areas was relatively higher than that of teachers in rural areas (except the SCL-90 factor “Somatization”). The results of the mean comparison were generally consistent with the effect size calculated in the subgroup analysis. The inconsistency (MD_western_ > MD_central_, M_western_ < M_central_) could be mainly explained by differences in sample size and the degree of fluctuation in the studies’ results. Based on subgroup analysis and mean comparisons, it could be found that, generally, teachers in the Eastern and Northeastern regions/urban areas have better mental health than those in the Western China and Central China regions/rural areas, respectively.

The linear regression model shown in Equation 3 was used to explore the relationship between the SCL-90 scores of primary and secondary school teachers and economic factors (per capita disposable income, per capita disposable income growth rate, and Engel’s coefficient) from a cross-temporal perspective. For models with large Variance Inflation Factor (VIF > 10), ridge regression was used as a supplement to assess whether an effective regression model exists (see Supplementary file S6). The results showed that the model was not effective for the SCL-90 score when the time lag was 0–3 years. For each condition, the value of *R*^2^ was much less than 1, indicating that the fitting degree of the regression model was not good, and the F-test results were not significant (*p*-value > 0.05), indicating that there did not exist a significant linear relationship between the selected independent and dependent variables (see Table 8; Supplementary file S6).

**Table 8:** Linear regression for SCL-90 factors (k = 153).

Factors	Lag 0		Lag 1		Lag 2		Lag 3	
	*R^2^*	*F*	*R^2^*	*F*	*R^2^*	*F*	*R^2^*	*F*
Somatization	0.062	0.393	0.128	0.878	0.110	0.743	0.152	1.078
Obsessive-compulsive	0.079	0.517	0.095	0.631	0.087	0.575	0.095	0.630
Interpersonal sensitivity	0.001	0.004	0.032	0.201	0.024	0.150	0.033	0.202
Depression	0.023	0.144	0.043	0.268	0.040	0.253	0.047	0.297
Anxiety	0.013	0.081	0.028	0.175	0.028	0.170	0.037	0.233
Hostility	0.019	0.117	0.034	0.211	0.031	0.195	0.036	0.222
Phobic anxiety	0.003	0.020	0.022	0.137	0.018	0.108	0.031	0.195
Paranoid ideation	0.015	0.088	0.036	0.224	0.025	0.157	0.036	0.223
Psychoticism	0.012	0.073	0.048	0.302	0.042	0.266	0.053	0.338
Total score	0.018	0.113	0.045	0.285	0.041	0.258	0.055	0.351

^*^p < 0.05, ^**^p < 0.01; k’, number of studies for subgroup (including valid mixed samples).

The overall results of multiple linear regression indicated that, from a cross-temporal perspective, the economic development level (described by per capita disposable income, per capita disposable income growth rate, and Engel’s coefficient) does not adequately explain the changing trends in primary and secondary school teachers’ mental health levels.

## 4 Discussion

From 1998 to 2021, the overall mental health level of primary and secondary school teachers decreased first and then slightly increased, showing an overall trend of slow decline, which is generally consistent with existing research findings (Wang et al., 2013; Wang et al., 2021; Wu, 2014; Xiao and Wu, 2018; Zhang, 2010; Zhao and Yang, 2015). This may be mainly related to the year-by-year increase in occupational stress. First, the social background has put forward higher requirements for individuals’ psychological adaptation levels. The enormous social changes brought about by reform and opening up have greatly accelerated the pace of social operations, and, to some extent, increased the difficulty of teachers’ psychological adaptation (Silbereisen et al., 2010; Xin and Chi, 2020). Second, professional characteristics pose a greater challenge to primary and secondary school teachers. Due to the young age of the students and the critical period for knowledge accumulation in primary and secondary school, their parents usually have higher expectations for primary and secondary school teachers. This high expectation leads to close attention, and the receiving of high-intensity social supervision brings more stress to teachers and triggers their job burnout (Sandmeier et al., 2022). Third, with the prevalence of the merit doctrine (Dong et al., 2023), some non-teaching tasks have taken up teachers’ already insufficient time, intensifying the troubles caused by social comparison (van Noord et al., 2025) and leading to exhaustion with no relief (Shen et al., 2025). Therefore, primary and secondary school teachers need to invest more psychological resources in emotional management during their specific educational and teaching practices. Over time, as their psychological resources continuously remain in a high-consumption state, a gradual decline in teachers’ mental health levels is a foreseeable result. The improvement in primary and secondary teachers’ mental health levels from 2012 to 2021 may be related to the effective implementation of the governing philosophy of the new generation of national leaders. Since 2012, China has continuously promoted a series of major livelihood reform measures for enhancing people’s wellbeing and promoting social equity and justice in order to effectively enhance people’s sense of gain, happiness, and security. At the 2018 National Education Conference, it was emphasized that it is necessary to continuously improve the treatment of teachers so that they can teach with peace of mind and enthusiasm. Under the continuous promotion of this guidance and these measures, the primary and secondary school teachers’ mental health has, to some extent, stopped worsening and started to improve.

Among the nine factors, somatization and coercion changed more obviously, and the factor values were higher, indicating that these two factors were more sensitive indicators of the mental health level of primary and secondary school teachers. The high somatization score reflects the strong correlation between psychology and physiology (Ku et al., 2025; Uzan, 2022). The feeling that the psychological pressure caused by work continues to rise and cannot be eliminated is often manifested by somatization symptoms such as headache, insomnia, gastrointestinal discomfort, etc. The high score of compulsion may be related to the nature of work (Tape, 2008). As primary and secondary school are important stages in laying the foundation for people’s outlook on life, teachers will more consciously practice “being a model for others,” resulting in a decline in the coordination of their “perfectionism consciousness.” Over time, teachers may develop a certain compulsion in life.

The poor mental health status of teachers in the Western China region may be due to long-term lagging economic development and difficult natural environment conditions. The problematic mental health level in the Central China region may be related to macro-regional policies as well as population structure. Compared with the significant development of China’s western region, development in the central region is relatively slow, and industrial structure transformation faces difficulties. The overall state of society has affected the educational ecosystem, and the negative experiences of social comparison have also influenced teachers’ mental health. Furthermore, the high population density has led to educational pressure in both the central and eastern regions, while the economic and social resources in the central region are relatively insufficient, having a more negative impact on teachers.

From a horizontal perspective, the mental health levels of primary and secondary school teachers in regions with higher economic development are higher than those in regions with lower economic development. From a longitudinal perspective, there is no statistically significant correlation in the time dimension according to the results of linear regression, indicating that the development of economic level has not directly led to a sustained improvement in the mental health level of primary and secondary school teachers. The contradiction between horizontal and longitudinal analyses indicates that there may be additional factors that influence primary and secondary school teachers’ mental health levels through a complex process. The primary purpose of China’s economic development is to improve people’s lives and enhance their sense of wellbeing. There is a discrepancy between the generally declining trend of primary and secondary school teachers’ mental health levels and China’s aim of economic development.

The complex and contradictory relationship between trends in primary and secondary school teachers’ mental health levels and economic development status is similar to the viewpoint of the “Easterlin paradox” (Easterlin, 1974). Psychological health is closely related to happiness (Klochko, 2025； Yu, 2022) and is regarded as a significant indicator in measuring subjective wellbeing (Das et al., 2020). Thus, referring to the viewpoint of “happiness-income puzzle” from the Easterlin paradox, this study proposes a viewpoint as follows, trying to provide a hypothetical theoretical explanation for this phenomenon.

Economic factors are highly salient social indicators, and people’s sensitivity to their changes in them is usually expressed as a concern about income (especially “property income”) (Guo, 2021). For teachers from regions with higher levels of economic development, as their income can meet their consumption needs to a greater extent, their subjective wellbeing is enhanced, which has a positive impact on their mental health (Behera et al., 2024; Mikucka et al., 2017; Zhang, 2021). In contrast, teachers in regions with relatively lower economic development may not only be limited in meeting their consumption needs but also experience a decrease in their sense of social equity due to perceived relative deprivation (Folger, 2021; Zhao and Zhao, 2024). Therefore, at a specific time point, there is a positive correlation between economic development and the mental health level of primary and secondary school teachers. It should be pointed out that in some studies’ suggestions and conclusions, “teachers in rural school are mentally healthier than urban teachers, as there are fewer mental health problems in rural areas and impoverished communities” (Fan and Fang, 2025; Xiao et al., 2025). However, in the majority of selected literature in this study, urban teachers had lower SCL-90 scores. Thus, the meta-analysis concluded that primary and secondary school teachers in urban areas have relatively better mental health.

In the short term, teachers’ high incomes maintain a good level of mental health; however, in the longer term, their mental health level will not significantly improve with rising living standards, outwardly trapped in a “time puzzle.” A possible reason is that the factors affecting the mental health of primary and secondary school teachers are quite complex and diverse. At a longer timescale, in addition to economy, more social factors such as the rise of core consumer prices (e.g., house prices and childcare) under the role of marketization (Long and Liang, 2019), the registered residence system, education level (Zhong and Yue, 2020) are added into the factors group that affects mental health, meanwhile the rapid development of society has also accelerated the superposition and evolvement of these factors. Thus, the originally existing positive correlation between economic development and mental health has been diluted or even dispelled.

It should be pointed out that some studies using cross-temporal meta-analysis suggest that economic development has a negative impact on teachers’ mental health, such as the one-way causal relationship between economic rewards and psychological factors such as coercion (Yang and You, 2017), or the improvement of residents’ consumption level and gross domestic product can both positively predict teachers’ mental health problems (Chen et al., 2024; Huang et al., 2024). These studies may not have serious errors in statistical analysis methods; however, they may have oversimplified the factors that influence teachers’ mental health, and their analysis and inference are, to some extent, not aligned with the historical orientation of “China’s economic development is for the well-being of the people.” This inconsistency between academic research and the social situation also highlights the diversity and complexity of the factors that may influence primary and secondary school teachers’ mental health, indicating that the evaluation and enhancement of primary and secondary school teachers’ mental health levels may be a complex, systemic issue.

Therefore, to improve primary and secondary school teachers’ mental health in short term, enhancing their income competitiveness and improving their socioeconomic status are effective measures. While in the long term, more measures may need to be introduced. From a micro perspective, School administrators can find ways to enhance teachers’ intrinsic motivation to maintain their mental health, such as popularizing training to enhance mental health literacy (Nalipay and Simon, 2023), enhancing psychological resilience (Agyapong et al., 2023), and sensitivity to diverse intrinsic rewarding stimuli (Blain et al., 2023). From a meso perspective in education policymaking, positive factors beyond socioeconomic status should be given full consideration, such as peer support (Song and Wu, 2022), psychological capital (You and Yao, 2023), and professional identity (Guo and Fu, 2024). Reducing occupational stress (Malik et al., 2025) and relieving occupational burnout (Huynh et al., 2023) can reduce the negative impact that it has on teachers’ sense of happiness. From a macro perspective, efforts should also be made at the social level, such as building social trust (Mikucka et al., 2017), promoting emotional wellbeing (Behera et al., 2024), and comprehensively enhancing the inclusive growth index (United Nations Conference on Trade and Development, 2025). Overall, continuously improving the mental health of primary and secondary school teachers is a systematic project that requires multiple measures, focusing on both various profession-related factors and broader societal improvement.

The study explores changes in the mental health levels of primary and secondary school teachers in China using a cross-temporal meta-analysis and discusses their correlation with social changes from an economic perspective. First, the study analyzes the relationship between primary and secondary school teachers’ mental health levels and economic development over a long period of time. The “time puzzle of mental health” hypothesis proposed in this study echoes the Easterlin paradox and, to some extent, enriches research in related fields. Second, as primary and secondary school teachers share commonalities with other teachers, the analysis and conclusions of this study can also serve as a reference for research on the mental health of teachers in other grades. Third, China is the largest developing country and has attracted worldwide attention for its rapid economic growth. Its successful attempts on the development path have also provided a model for other developing countries to learn from. Thus, the “time puzzle of mental health” hypothesis has a certain degree of representativeness and generalizability and can provide a new perspective and a reference for more developing countries to explore the changes in the mental health of primary and secondary school teachers and make appropriate interventions.

## 5 Limitations and the future prospects

In this study, the discussion of primary and secondary school teachers’ mental health levels and their changing trends was based on an analysis of the SCL-90 scale. As the factors the SCL-90 measures are all negative psychological qualities, and the validity period of the SCL-90 results does not well fit adequately capture the variability of mental health status, as well as the mainstream Dual-Factor Model of Mental Health (DFM), the limitations of this scale may affect the research results. However, this study presents an opportunity for the future research to conduct an analysis from the perspective of positive psychology and to introduce factors such as mental health literacy and psychological capital into the research. The conclusion of meta-analysis largely depends on the selected data. In this study, fitting a curve and subgroup heterogeneity tests were used to evaluate the influence of density and absent data, and consensus was reached during the analysis of extreme values. As SCL-90 cannot trace mental status in the past, interviews and existing tracking investigations may be suitable as supplements.

Additionally, this study only examined how social changes affect primary and secondary school teachers’ mental health levels from an economic perspective. With consideration of the complexity and systematic nature of primary and secondary teachers’ mental health, the analysis can be extended to examine cultural concepts, population structure, and other aspects to further test the ecological validity of the conclusion. This study used data from China as samples, aiming to align with China’s policy of building the leading country in education and the historical orientation of “developing the economy for the people’s well-being.” Therefore, the particularity of the sample may to some extent affect the ecological validity. For the future studies, a wider sample source can be introduced to further improve the conclusion’s universality.

## 6 Conclusion

From 1998 to 2021, the overall mental health of primary and secondary school teachers first decreased and then slightly increased. There are significant differences among primary and secondary school teachers’ mental health levels in different economic regions, and teachers from regions with better economic development have higher mental health levels. The primary and secondary school teachers in urban areas have relatively better mental health levels than teachers in rural areas. From a cross-temporal perspective, the level of economic development cannot adequately explain the changing trend in primary and secondary school teachers’ mental health. The mental health of primary and secondary school teachers is a complex issue, and its effective improvement may require systematic consideration of multiple factors within the profession itself, the education system, and society as a whole, as well as both short-term and long-term measures. The “time puzzle of mental health” hypothesis proposed by this study echoes the Easterlin paradox, to some extent, enriches research in related fields, and provides a new theoretical perspective with reference value for explaining the relationship between economic development and primary and secondary school teachers’ mental health levels.

## Data Availability

The original contributions presented in the study are included in the article/Supplementary material, further inquiries can be directed to the corresponding author.

## References

[ref1] Agyapong B. Chishimba C. Wei Y. Dias R. L. Eboreime E. Msidi E. . (2023). Improving mental health literacy and reducing psychological problems among teachers in Zambia: protocol for implementation and evaluation of a Wellness4Teachers email messaging program. JMIR Res. Protoc. 12:e44370. doi: 10.2196/44370 36877571 PMC10028515

[ref2] Barker T. H. Hasanoff S. Aromataris E. Stone J. C. Leonardi-Bee J. Sears K. . (2026). The revised JBI critical appraisal tool for the assessment of risk of bias for analytical cross-sectional studies. JBI Evid Synth. 24, 401–408. doi: 10.11124/JBIES-24-00523, 40521701

[ref3] Behera B. K. Mishra S. R. Jena L. K. Pradhan R. K. (2024). Socioeconomic determinants of happiness: empirical evidence from developed and developing countries. J. Behav. Exp. Econ. 109:102187. doi: 10.1016/j.socec.2024.102187

[ref4] Blain B. Pinhorn I. Sharot T. (2023). Sensitivity to intrinsic rewards is domain general and related to mental health. Nat. Mental Health 1, 679–691. doi: 10.1038/s44220-023-00116-x, 38665692 PMC11041740

[ref5] Borenstein M. Hedges L. V. Higgins J. P. T. Rothstein H. R. (2013). Introduction to meta-Analysis. Beijing (China): Science Press.

[ref6] Chen Z. Yu X. Yu G. (2024). Features, influencing factors, and development trends of mental health problems among secondary school teachers. Renmin Univ. China Education J. 3, 144–155. doi: 10.12451/202406.00555

[ref7] Cohen J. (1977). Statistical power Analysis for the Behavioral Sciences. New York (USA).

[ref8] Das K. V. Jones-Harrell C. Fan Y. Ramaswami A. Orlove B. Botchwey N. (2020). Understanding subjective well-being: perspectives from psychology and public health. Public Health Rev. 41:25. doi: 10.1186/s40985-020-00142-5, 33292677 PMC7678314

[ref9] Dong Z. W. Cao Y. Hou Y. B. Dai Y. W. (2023). The role of career stress and resilience in the relationship between social comparison and mental health in primary and middle school teachers. Chin. J. Health Psychol. 31, 876–881. doi: 10.13342/j.cnki.cjhp.2023.06.016

[ref10] Easterlin R. A. (1974). “Does economic growth improve the human lot? Some empirical evidence,” in Nations and Households in Economic Growth: Essays in Honor of Moses Abramovitz, eds. David P. A. Reder M. W. (New York: Academic Press), 89–125.

[ref11] Fan X. Fang J. (2025). Strategic organizational support for stress and burnout: insights from rural teachers’ stress profiles. Front. Psychol. 16:1580573. doi: 10.3389/fpsyg.2025.1580573, 41000518 PMC12457424

[ref12] Fan H. Y. Li J. J. Zhao M. L. Li H. (2016). Mental health of kindergarten teachers: a meta-analysis of studies based on the SCL-90. Adv. Psychol. Sci. 24, 9–20. doi: 10.3724/SP.J.1042.2016.00009

[ref13] Folger R. (2021). Reformulating the preconditions of resentment. J. Moral Psychol. 15, 45–67.

[ref14] Guo W. (2021). Expanding domestic demand urgently requires increasing residents' property income. China Finance 3, 68–69.

[ref15] Guo B. Fu Y. X. (2024). The relationship between self-efficacy and professional identity of primary and secondary school music teachers in Western China: the mediating role of leaving intention and work commitment. J. Capital Normal Univ. 4, 183–191.

[ref16] Harding S. Kidger J. Bryant L. Bloor K. Brockman R. (2019). Is teachers' mental health and wellbeing associated with students' mental health and wellbeing? J. Affect. Disord. 253, 460–466. doi: 10.1016/j.jad.2019.03.046 30189355

[ref17] Huang X. Jin J. Yu G. (2024). Features, influencing factors, and development trends of mental health problems among Chinese teacher groups. Renmin Univ. China Education J. 3, 168–180. doi: 10.12451/202406.00554

[ref18] Huynh H. V. Proeschold-Bell R. J. Sohail M. M. Nalianya M. Wafula S. Amanya C. . (2023). What processes or key components do teachers attribute to their well-being? A cross-cultural qualitative study of teacher well-being in Cambodia, Kenya, and Qatar. Psychol. Sch. 60, 4967–4987. doi: 10.1002/pits.23043

[ref19] Klochko T. (2025). “Mental health as a factor of individual’s subjective well-being,” in Socioeconomic Determinants of Happiness: Empirical Evidence from Developed and Developing Countries, eds. Beaumont L. K.. (Hershey: IGI Global), 139–159.

[ref20] Ku X. Jyung M. Kim J. H. Choi I. (2025). Sound mind, sound body, or vice versa? Mind-body beliefs shape health behaviors. Appl. Psychol. Health Well Being 17:e12617. doi: 10.1111/aphw.12617, 39494481

[ref21] Liu H. F. Chen S. J. Sun J. Y. Wang Z. J. Ke Z. Zhang M. K. (2023). The theoretical thinking and key directions for building a strong education nation. China Educ. Technol. 10, 1–17.

[ref22] Long F. Liang X. Q. (2019). The structural influence of social economic status and family core consumption pressure on Chinese family happiness——based on the empirical research of CSS2013. Soc. Sci. Res. 2, 94–103.

[ref23] Malik K. Reddy S. Shilla Y. A. Khanna A. Kaurr R. R. Boban A. (2025). Implementing psychosocial interventions for teachers’ mental health: protocol for integrating scoping review with teachers lived experiences in LMICs. PLoS One 20:e0317351. doi: 10.1371/journal.pone.0317351, 39869609 PMC11771928

[ref24] Mikucka M. Sarracino F. Dumitru S. (2017). When does economic growth improve life satisfaction? Multilevel analysis of the roles of social trust and income inequality in 46 countries, 1981–2012. World Dev. 93, 447–459. doi: 10.1016/j.worlddev.2017.01.002

[ref25] Nalipay M. J. N. Simon P. D. (2023). Teachers are frontliners too: promoting mental health literacy among teachers in low-and middle-income countries. Asian J. Psychiatr. 81:103407. doi: 10.1016/j.ajp.2022.103407, 36525888

[ref26] Page M. J. McKenzie J. E. Bossuyt P. M. Boutron I. Hoffmann T. C. Mulrow C. D. . (2021). The PRISMA 2020 statement: an updated guideline for reporting systematic reviews. BMJ 372:n71. doi: 10.1136/bmj.n71, 33782057 PMC8005924

[ref27] Sandmeier A. Baeriswyl S. Krause A. Muehlhausen J. (2022). Work until you drop: effects of work overload, prolonging working hours, and autonomy need satisfaction on exhaustion in teachers. Teach. Teach. Educ. 118:103843. doi: 10.1016/j.tate.2022.103843

[ref28] Shen Z. Lan R. Su X. Lian R. Zhang Y. (2025). The relationship between extra-administrative workload, emotional exhaustion, and work engagement of primary and secondary school teachers: based on multilevel linear model analysis. Behav. Sci. 15:1405. doi: 10.3390/bs15101405, 41153195 PMC12561797

[ref29] Silbereisen R. K. Pinquart M. Tomasik M. J. (2010). “Demands of social change and psychosocial adjustment: results from the Jena study,” in Social change and human Development: Concept and Results, eds. Silbereisen R. K. Chen X. (Thousand Oaks: SAGE Publications), 125–147.

[ref30] Song H. Wu Y. C. (2022). Effects of social support from colleagues on teachers' subjective wellbeing in compulsory education in Western China. J. South China Normal Univ. 6, 49–60.

[ref31] Tape W. (2008). Exploring the sense of Coherence and Psychological Symptomatology of Teachers in under-Resourced Schools in Cape Town [Doctoral Dissertation]. Cape Town: University of Cape Town.

[ref32] Tong H. J. (2010). A research of twenty years’ vicissitude: SCL-90 and its norm. Psychol. Sci. 33, 928–930+921. doi: 10.16719/j.cnki.1671-6981.2010.04.022

[ref33] Twenge J. M. Campbell W. K. Freeman E. C. (2012). Generational differences in young adults' life goals, concern for others, and civic orientation, 1966–2009. J. Pers. Soc. Psychol. 102, 1045–1062. doi: 10.1037/a0027408, 22390226

[ref34] Twenge J. M. Konrath S. Foster J. D. Campbell W. K. Bushman B. J. (2008). Egos inflating over time: a cross-temporal meta-analysis of the narcissistic personality inventory. J. Pers. 76, 875–902. doi: 10.1111/j.1467-6494.2008.00507, 18507710

[ref35] United Nations Conference on Trade and Development (2025) Beyond GDP: New data reveal Persistent gaps and progress in Inclusive Growth (Inclusive Growth Report 2025) United Nations Available online at: https://unctad.org/publication/inclusive-growth-report-2025 (Accessed February 7, 2026).

[ref36] Uzan P. (2022). Mind-body Entanglement: Theory and Therapies. Cham: Springer.

[ref37] van Noord J. Spruyt B. Van Droogenbroeck F. Kuppens T. (2025). Caught between ideology and self-interest: subjective social status and meritocratic beliefs shape whether people perceive, feel anger about, and want to change economic conflict. Br. J. Sociol. 76, 566–577. doi: 10.1111/1468-4446.13185, 39916323

[ref38] Wang Z. Y. (1984). Symptom checklist-90 (SCL-90). Shanghai Arch. Psychiatry 2, 68–70.

[ref39] Wang H. B. Chen N. Chen F. (2013). A cross-temporal meta-analysis of the mental health status of primary and secondary school teachers. Shanghai Res. Educ. 2, 41–45. doi: 10.16194/j.cnki.31-1059/g4.2013.02.008

[ref40] Wang Q. Yu Y. Yang Y. Yao B. J. (2021). Mental health status of primary and secondary school teachers in China from 2010 to 2020: a meta-analysis based on SCL-90 scores. J. Hotan Prefectural Educ. College. 40, 46–53.

[ref41] Wu H. Y. (2014). An analysis of SCL-90 test results of regular middle school teachers in recent fourteen years. Chin. J. Clin. Psych. 22, 702–706. doi: 10.16128/j.cnki.1005-3611.2014.04.031

[ref42] Xiao S. Wang C. Zhang L. Xiang X. Cortright R. E. Pednekar R. S. . (2025). Association between demographic factors and mental health outcomes among primary and secondary school teachers in southeast of China: a cross-sectional study. BMC Psychol. 14:11. doi: 10.1186/s40359-025-03769-8, 41331482 PMC12776953

[ref43] Xiao T. Wu Z. H. (2018). Changes in the mental health status of rural teachers in China (1991–2014): a cross-temporal meta-analysis. Educ. Sci. Res. 8, 69–77.

[ref44] Xin Z. Q. Chi L. P. (2008). Cross-temporal meta-analysis: review of research on psychologica development in social change. J. East China Normal Univ. 26, 44–51. doi: 10.16382/j.cnki.1000-5560.2008.02.001

[ref45] Xin Z. Q. Chi L. P. (2020). Trends in changes of contemporary Chinese mental health. People's Tribune. 1, 46–50.

[ref46] Yang R. J. You X. Q. (2017). Advancing the effort-reward imbalance theory: the effect of economic rewards on teachers' mental health. Acta Psychol. Sin. 49, 1184–1194. doi: 10.3724/SP.J.1041.2017.01184

[ref47] You Y. S. Yao J. H. (2023). Principal-teacher management communication and teachers’ knowledge sharing: the mediating role of teachers’ psychological empowerment and psychological capital. J. Educ. Stud. 19, 118–129. doi: 10.14082/j.cnki.1673-1298.2023.05.010

[ref48] Yu G. L. (2022). A new interpretation of mental health: from the perspective of well-being. J. Beijing Normal Univ. 1, 72–81.

[ref49] * Yu Q. M. Li L. Zhou B. Liao J. (2015). Mental health of primary and secondary teachers in Yunnan Province. Chin. J. Health Psychol. 23, 201–204. doi: 10.13342/j.cnki.cjhp.2015.02.014

[ref50] Zhang Y. L. (2010). The meta-analysis of mental health about primary and junior school teachers (Master’s Thesis, Hebei Normal University).

[ref51] Zhang T. W. (2021). Relationship between income level, income gap and subjective happiness: empirical analysis based on CGSS data of six provinces in 2017. Areal Res. Development. 40, 31–36. doi: 10.3969/J.ISSN.1003-2363.2021.03.006

[ref52] * Zhang L. L. Xiao H. Q. Zheng H. B. Xiao Y. N. Chen M. Y. Chen D. L. . (2013). Mental health of graduating class teachers of primary and secondary schools in Luoding city. Chin. J. Health Psychol. 21, 1059–1061. doi: 10.13342/j.cnki.cjhp.2013.07.010

[ref53] Zhao Y. L. Yang X. L. (2015). Changes in mental health of primary and secondary school teachers in China from 1991 to 2010. Chin. J. Sch. Health. 36, 556–559+562. doi: 10.16835/j.cnki.1000-9817.2015.04.026

[ref54] Zhao X. D. Zhao D. J. (2024). Research on the impact of income inequality on residents’ happiness. Scientific Decision Making 3, 1–20. doi: 10.3773/j.issn.1006-4885.2024.03.001

[ref55] Zhong C. Yue X. M. (2020). Why does economic growth fail to bring increase of happiness? A review of factors influencing subjective well-being. Nankai Econ. Stud. 4, 24–45. doi: 10.14116/j.nkes.2020.04.002

