# Supplementary material for: The changes in primary and secondary school teachers’ mental health level according to SCL-90 and its economic dissection—a meta-analysis with evidence from China
Source: Front Psychol. 2026 Jul 29;17:1815346. doi: 10.3389/fpsyg.2026.1815346 (PMC13461991; doi:10.3389/fpsyg.2026.1815346)
Supplement: Supplementary file 1 [file Data_Sheet_1.PDF]

Supplementary file 1. Information of literature coding

Study name	Sample size	Research year	Publication	Economic regions	Urban/rural areas
Zhu, X. L. et al. (2000)	312	1998	C	C	R
Wang, Y. Z. (2000)	158	1998	C	W	R
Xu, H. (2000)	308	1998	C	E	U
Xiao, S. R. et al. (2005)	423	2000	C	C	R
Wu, Z. X. (2002)	167	2000	NC	E	/
Li, C. L. (2002)	154	2000	C	W	U
Chen, J. F. et al. (2002)	52	2000	C	E	U
Zhang, Q. (2002)	230	2000	C	W	U
Dong, W. J. (2002)	178	2001	NC	W	R
Yu, X. X. (2003)	182	2001	NC	W	/
Yang, S. Y. et al.(2003)	341	2001	C	W	R
Zhang, D. X. (2003)	412	2001	NC	E	M
Qiu, L. (2003)	1321	2001	C	E	M
Zhang, S. F. et al. (2003)	381	2001	NC	C	M
Li, J., Liu et al. (2003)	187	2002	NC	/	U
Sheng, S. Z. et al. (2004)	32	2002	C	E	/
Feng, W. et al. (2004)	243	2002	C	/	/
Li, J. Q. (2004)	116	2002	C	C	U
Ding, F. Q. (2004)	631	2002	C	W	R
Shen, Z. R. et al. (2005)	309	2002	C	/	/
Wang, C. et al. (2004)	1016	2002	NC	/	/
Shen, Z. D. et al. (2004)	39	2002	C	E	/
Wang, X. Q. et al. (2004)	271	2002	C	C	/
Li, B. Z. et al. (2004)	566	2002	C	E	M
Yu, J. (2004)	152	2002	D	NE	/
Li, J. (2004)	160	2002	NC	E	M
Li, F. Q. (2004)	176	2002	NC	W	M
Xiang, Z. Q. et al. (2004)	400	2002	C	E	U
Liu, S. Q. et al. (2004)	115	2002	NC	E	/
Wang, J. Z. et al. (2004)	924	2002	C	E	U
Lin, H. L. (2004)	926	2002	C	E	M
Wang, M. H. et al. (2004)	210	2002	NC	E	U
Chen, Q. Y. et al. (2004)	258	2002	C	W	R
Li, J. et al (2003)	796	2003	C	E	U
Liu, B. et al. (2005)	1187	2003	NC	W	U
Wang, L. J. (2005)	902	2003	C	C	M
Wang, L. J. (2005)	438	2003	NC	C	R
Zhang, Y. F. (2005)	1000	2003	NC	E	M

Wu, A. H. (2005)	822	2003	D	E	U
Cheng, M. (2005)	118	2003	D	W	U
Liao, Q. M. et al. (2005)	121	2003	NC	W	R
Xiao, S. B. et al. (2005)	291	2003	NC	E	M
Lin, X. (2005)	274	2003	NC	E	M
Ma, H. X. et al. (2004)	78	2003	C	C	/
Wei, K. X. et al. (2006)	280	2003	NC	E	M
Zhou, C. E. et al. (2006)	521	2004	C	E	M
Ren, Q. P. (2006)	476	2004	NC	C	R
Chen, S. (2006)	107	2004	NC	C	R
Cai, Y. Q. (2006)	258	2004	D	NE	U
Deng, W. G. et al. (2006)	245	2004	NC	C	M
Pan, X. F. et al (2006)	245	2004	NC	C	M
Wang, Z. M. (2006)	918	2004	C	E	/
Zhang, Y. Z. et al. (2006)	212	2004	C	C	R
Zhou, X. Q. et al. (2006)	147	2004	C	C	U
Gui, Q. R. (2006)	500	2004	D	E	U
Fang, H. L. et al. (2007)	5055	2004	C	W	/
Yang, L. J. et al. (2006)	520	2004	C	E	M
Lin, H. L. (2005)	97	2004	C	E	U
Long, Q. Z. (2006)	269	2004	C	W	M
Zhang, W. F. (2006)	288	2004	C	E	U
Luo, X. L. (2006)	146	2004	C	C	R
Wang, W. Z. et al. (2007)	564	2005	C	/	/
Du, L. J. (2007)	424	2005	NC	C	M
Zheng, Y. Z. (2007)	144	2005	NC	E	U
Dong, A. G. et al. (2007)	279	2005	C	W	R
Ding, Y. L. (2007)	272	2005	D	W	U
Li, M. Y. (2007)	556	2005	D	NE	M
Wang, H. (2005)	216	2005	D	E	R
Tang, Z. Q. et al. (2007)	343	2005	C	E	U
Yang, Y. S. et al. (2007)	231	2005	NC	W	/
Chen, X. Y. et al. (2007)	426	2005	NC	E	/
Liu, X. W. (2007)	251	2006	NC	E	R
Zhou, L. et al. (2008)	216	2006	NC	W	R
Wei, Y. Y. et al. (2008)	1644	2006	NC	C	U
Sun, Y. Q. (2008)	364	2006	NC	NE	M
Ju, X. M. (2008)	460	2006	NC	W	U
Liu, Y. H. (2007)	618	2006	D	W	U
Liu, W. J. (2007)	480	2006	D	W	R
Zhou, Y. X. (2008)	1664	2006	D	C	U

Yu, D. J. et al. (2008)	341	2006	C	NE	U
Li, F. Y. et al. (2008)	1362	2006	NC	W	/
Wei, Q. (2009)	97	2006	C	W	/
Liu, Y. L. (2008)	125	2006	NC	E	M
Zhao, M. Y. (2007)	430	2006	C	C	U
Wang. Z. C. et al. (2008)	146	2006	NC	W	R
Li, F. Y. et al. (2008)	1362	2007	C	W	U
Zhang, X. (2009)	542	2007	NC	W	R
Huang, X. Y. et al. (2009)	653	2007	NC	E	R
She, L. Z. et al. (2009)	138	2007	NC	C	R
Chen, D. R. (2009)	462	2007	D	C	U
Hu, M. H. (2007)	325	2007	D	C	R
Liu, W. (2009)	185	2007	D	W	U
Huang, X. Y. et al. (2009)	236	2007	NC	E	/
Li, C. J. (2008)	1140	2007	NC	C	M
Xing, Q. (2010)	339	2008	NC	E	U
He, S. D. (2010)	810	2008	C	W	U
Hu, W. P. et al. (2010)	3352	2008	C	C	U
Wang, L. et al. (2010)	87	2008	C	C	R
Liu, D. F. et al. (2010)	82	2008	NC	C	U
Wang, W. Y. et al. (2010)	589	2008	NC	E	M
Liu, J. M. (2010)	777	2008	D	W	/
Li, J. P. (2010)	512	2008	D	NE	M
Ye, F. H. et al. (2011)	640	2009	C	C	U
Lu, H. L. et al. (2011)	456	2009	C	C	R
Zhang, Y. Q. (2010)	329	2009	D	W	R
Chen, L. J. et al. (2011)	236	2009	NC	E	R
Zhao, H. L. (2011)	225	2009	NC	C	R
Sun, Z. (2011)	273	2009	NC	C	R
Song, Z. H. et al. (2011)	545	2009	C	E/W	/
Zhang, L. P. (2010)	78	2009	NC	C	R
Wang, D. C. (2012)	226	2010	NC	NE	U
Peng, Y. H. et al. (2012)	423	2010	C	C	R
Wang, X. M. et al. (2012)	205	2010	NC	W	U
Liu, Y. et al. (2012)	1143	2010	C	W	/
Luo, Y. et al. (2013)	398	2010	NC	C	M
Hu, X. et al. (2012)	183	2010	NC	NE	/
Gao, M. S. (2011)	8015	2011	NC	/	R
Pan, K. Q. et al. (2013)	362	2011	NC	W	U
Yu, H. T. (2013)	187	2011	D	NE	U
Wang, Z. C. (2013)	405	2011	D	W	U

Chen, Z. M. (2013)	1232	2011	NC	E	M
Zhang, L. L. et al. (2013)	630	2012	C	E	R
Su, B. Y. et al. (2013)	624	2012	NC	E	U
Hu, H. Y. et al. (2014)	362	2012	C	E	U
Han, M. M. et al. (2014)	1321	2012	NC	W	U
Wu, B. R. (2014)	456	2012	D	C	U
Wang, Y. M. et al. (2014)	223	2012	C	W	U
Wu, H. Y. (2014)	410	2012	NC	W	U
Zhang, L. et al. (2014)	590	2012	C	E	R
Wang, Y. C. et al. (2014)	509	2013	NC	NE	/
Yu, Q. M. et al. (2015)	460	2013	C	W	M
Chen, Z. L. ET AL. (2015)	1260	2013	NC	E	U
Zhang, P. et al. (2015)	274	2013	NC	W	R
Yang, H. B. et al. (2015)	126	2013	NC	W	U
Zheng, Y. J. et al. (2015)	252	2013	C	E	U
Kang, Y. W. et al. (2015)	256	2013	NC	W	M
Zhou, H. M. (2015)	245	2013	NC	E	U
Zhang, X. Q. et al. (2015)	86	2013	NC	W	R
Guan, T. et al. (2015)	2340	2014	NC	NE	U
Fan, X. Y. (2016)	276	2014	D	C	R
Zhang, X. X. (2016)	752	2014	NC	W	U
Zhang, W. et al. (2017)	189	2015	NC	W	M
Ma, C. F. (2018)	438	2016	C	W	R
Wu, X. M. (2018)	335	2016	D	E	U
Li, X. (2018)	216	2016	NC	W	/
Dong, Y. X. (2019)	281	2018	D	NE	U
Deng, X. B. et al. (2020)	4223	2018	NC	W	U
Yang, Y. et al. (2019)	123	2018	NC	E	U
Yue, C. Z. et al. (2021)	462	2019	NC	W	U
Gao, T. L. (2021)	912	2019	NC	W	R
Zhang, B. et al. (2022)	1793	2020	NC	W	U
Jiang, Y. et al. (2022)	255	2020	NC	C	U
Fang, G. X. et al. (2023)	1025	2021	C	W	R

Note: Publication (C: core journal, NC: non-core journal, D: dissertation). Economic regions (E: eastern China, W: western China, NE: northeastern China, C: central China). Urban/rural (U: urban area, R: rural area).

Supplementary file 2. Literatures included in meta-analysis (k=153)

Cai Y. Q. (2006). *Current situation and countermeasures of mental health of primary and secondary school teachers* (Master's thesis, Northeast Normal University).

Chen D. R. (2009). *Mental health and its relationship with occupational stress, social support study of secondary school teachers in Zhengzhou City* (Master's thesis, Capital Normal University).

Chen J. F., Chen L. H., & Lin Y. Q. (2002). Assessment and analysis of primary school teachers by SCL-90. *China Journal of Health Psychology*, (03), 190–191. doi:10.13342/j.cnki.cjhp.2002.03.015

Chen L. J., Huang X. Y., Huang D. Y., Zhu C., & Wu Y. F. (2011). Investigation and analysis on mental health status of secondary school teachers. *China Modern Doctor*, 49(20), 9–10+23.

Chen Q. Y., Qian M., & Guo Y. (2004). Analysis of teachers' mental health status and influencing factors in western ethnic regions. *Journal of Southwest Minzu University (Humanities and Social Sciences Edition)*, (09), 21–25.

Chen S. (2006). Paying attention to teachers' mental health: An investigative analysis of teachers' mental health. *Journal of Tongling College*, (04), 125–127+129. doi:10.16394/j.cnki.34-1258/z.2006.04.053

Chen X. Y., & Guo H. W. (2007). Investigation and analysis on the mental health status of primary and secondary school teachers. *Sichuan Mental Health*, (03), 174.

Chen Z. L., Chen Z. M., Xiao Y. N., Chen D. L., & Wu F. C. (2015). Analysis of mental health status of primary and secondary school teachers in Luoding City. *Sichuan Mental Health*, 28(05), 453–457.

Chen Z. M. (2013). Psychological health investigation of primary and secondary school teachers in Luoding City. *China Modern Medicine*, 20(20), 172–173.

Cheng M. (2005). *Investigation, research and countermeasures on the mental health status of middle school teachers in Kunming* (Master's thesis, Yunnan Normal University).

Deng W. G., Pan X. F., & He H. Y. (2006). Correlation analysis between school interpersonal atmosphere and teachers' mental health. *Journal of Educational Science of Hunan Normal University*, (06), 78–81.

Deng X. B., Tian L. Y., & Ji P. (2020). Investigation on the mental health status of compulsory education stage teachers and its influencing factors — Taking teachers in District A of Chengdu as an example. *Journal of Chengdu Normal University*, 36(11), 7–14.

Ding F. Q. (2004). Mental health of primary and secondary school teachers in Ningxia. *China Journal of Health Psychology*, (06), 408–410. doi:10.13342/j.cnki.cjhp.2004.06.004

Ding Y. L. (2007). *Investigation of Liuzhou junior middle school teachers' mental health and the countermeasures against it* (Master's thesis, Guangxi Normal University).

Dong A. G., & Liu A. J. (2007). Investigation and analysis on mental health status of rural junior middle school teachers in Shangluo City. *China Journal of Health Psychology*, (06), 552–554. doi:10.13342/j.cnki.cjhp.2007.06.034

Dong W. J. (2002). Psychological Health Survey on Primary & Secondary School Teachers in Underdeveloped Areas. *Journal of Shaanxi University of Technology(Social Sciences)*, (04), 70–74.

Dong Y. X. (2019). *The impact and enhancement of social support and coping styles on the mental health of primary and secondary school teachers: The mediating role of mental health literacy* (Master's thesis, Beihua University).

Du L. J. (2007). An Analysis of the State of the Teachers Mental Health of in Primary and Middle Schools. *Journal of Xinxiang University (Social Science Edition)*, (03), 138–140.

Fan X. Y. (2016). *The relationship among occupational stress, resilience and mental health of rural primary and secondary*

school teachers (Master's thesis, Yangzhou University).

Fang G. X., Zhou X. F., Xin Y., Li M., Li F., Zhang W. W., Li B., & Wang Y. (2023). Mental health of primary and secondary school teachers in the remote mountain areas. *Medicina (Kaunas, Lithuania)*, 59(5), 971. doi:10.3390/medicina59050971

Fang H. L., Zhang G. Q., Yang J. X., Zhang L., Song G. M., Huang L. S., …, & Qin M. (2007). Comparison of mental health levels in teachers from middle-primary schools and colleges in western areas. *Chinese Journal of Tissue Engineering Research*, (39), 7887–7890.

Feng W., & Zhang M. F. (2004). Psychological health of 243 national primary and secondary school key teachers. *Journal of Shanghai Educational Research*, (04), 28–30. doi:10.16194/j.cnki.31-1059/g4.2004.04.013

Gao M. S. (2011). Mental health status of rural primary and secondary school teachers in central and western China. *Journal of Beijing Institute of Education (Natural Science Edition)*, 6(02), 8–14.

Gao T. L. (2021). The current situation and countermeasures of rural middle school teachers' mental health in the context of the new era — A case study of rural middle school teachers in northwest Yunnan. *Survey of Education*, 10(03), 78–81. doi:10.16070/j.cnki.cn45-1388/g4s.2021.03.024

Guan T., & Yang D. (2015). Investigation on mental health status of primary and secondary school teachers in Shenyang and analysis of group intervention results. *Medical Journal of Chinese People's Health*, 27(19), 81–82+91.

Gui Q. R. (2006). *Research on current situation of mental health of primary and secondary school teachers in Nanjing and its countermeasures* (Master's thesis, Nanjing Normal University).

Han M. M., Wan H., Zhao Z. R., & Liu J. W. (2014). Study on the relationship between teachers' mental health and work ability. *Journal of Xinjiang Medical University*, 37(11), 1515–1518.

He S. D. (2010). Mental health problems and countermeasures of primary and secondary school teachers in Dazhou City, Sichuan Province. *Fujian Tribune (A Economics & Sociology Monthly)*, (02), 92–93.

Hu H. Y., Zhang L. H., & Li F. J. (2014). Investigation on mental health of primary and secondary school teachers. *China Journal of Health Psychology*, 22(09), 1342–1344. doi:10.13342/j.cnki.cjhp.2014.09.026

Hu M. H. (2007). *Study on job burnout and mental health of middle school teachers in the rural area of Jingzhou district, Jingzhou City* (Master's thesis, Central China Normal University).

Hu W. P., Ma Y. X., Jiao L. Y., & Wang H. Y. (2010). Investigation on mental health status of primary and secondary school teachers in Shanxi Province. *Theory and Practice of Education*, 30(10), 57–60.

Hu X., & Zhang H. J. (2012). Analysis of influencing factors on the mental health status of junior high school teachers in Liaoning Province. *Shanxi Medical Journal*, 41(06), 532–534.

Huang X. Y., Chen L. J., Zhu C., & Wu Y. F. (2009). A study on the correlation between personality traits and mental health of primary and secondary school teachers. *Contemporary Medicine*, 15(24), 10–12.

Huang X. Y., Chen L. J., Zhu C., & Wu Y. F. (2009). Investigation and study on mental health status of primary school teachers. *Contemporary Medicine*, 15(21), 157–159.

Jiang Y., Zhu L., Tang X. L., Xu S. L., & Tong Q. H. (2022). The mediating role of social support in the relationship between job burnout and mental health of middle school teachers during the normalization period of epidemic prevention and control. *Anhui Medical Journal*, 43(04), 468–472.

Ju X. M. (2008). An investigation and analysis on the mental health status of primary and secondary school teachers in the context of the new curriculum: Taking seven primary and secondary schools in Chuxiong City as an example. *Journal of Chuxiong Normal University*, 23(11), 90–96.

Kang Y. W., Kang X. W., & Wang D. F. (2015). A survey on mental health of primary school teachers in Tibet. *Tibet's Science*

and Technology, (08), 30–33.

Li B. Z., Wang K., & Li H. W. (2004). A study on mental health of middle and primary school teachers. *Studies of Psychology and Behavior*, (01), 360–363+372.

Li C. J. (2008). Investigation and research on mental health of primary and secondary school teachers in Puyang City. *Modern Economic Information*, (08), 125–126.

Li C. L. (2002). Mental health of primary school teachers in Nanning City and its influencing factors. *Chinese Journal of School Health*, (02), 172–173.

Li F. Q. (2004). Investigation on mental health status of primary school teachers in Chifeng City, Inner Mongolia. *Journal of Zhaowuda Mongolian Teachers College (Chinese Philosophy and Social Sciences Edition)*, (04), 92–94. doi:10.13398/j.cnki.issn1673-2596.2004.04.042

Li F. Y., Liu J. W., Liu B., & Lian Y. L. (2008). Study on mental health and work ability of middle and elementary school teachers in Urumqi. *Chinese Journal of Public Health*, 24(08), 939–941.

Li F. Y., Liu J. W., Liu B., & Lian Y. L. (2008). Study on the mental health status of middle and elementary school teachers in Urumqi. *Journal of Xinjiang Medical University*, 31(01), 4–7. doi:10.3969/j.issn.1009-5551.2008.01.002

Li J., & Chen T. (2004). Investigation and analysis on mental health status of 160 primary school teachers. *Chinese Journal of School Doctor*, (02), 154–155.

Li J., Chen T., Chu X. Q., Gao C., & Wang D. Q. (2003). A comparative study on mental health status of urban and rural primary and secondary school teachers in Jining City. *Chinese General Practice*, (12), 1012–1013.

Li J., Liu G. Y., Sui G. Y., Zhai M., & Song Y. W. (2003). Relationship between interpersonal relationship and mental health of primary and secondary school teachers. *Journal of Jining Medical University*, (03), 8–9.

Li J. P. (2010). *A comparative study on Jilin urban and rural middle school P.E. teachers' mental health and its influencing factors* (Master's thesis, Shenyang Sport University).

Li J. Q. (2004). Study of Mental Health Level of Primary and Secondary School Teachers in Xinxiang. *China Journal of Health Psychology*, (04), 292–293+243. doi:10.13342/j.cnki.cjhp.2004.04.023

Li M. Y. (2007). *The Yanbian area junior high school teachers' mental health research* (Master's thesis, Yanbian University).

Li X. (2018). A comparative study on the mental health levels of special education teachers and general primary school teachers in Sichuan Province. *Journal of Suihua University*, 38(04), 120–124.

Liao Q. M., Yang J., & Rao J. Y. (2005). A sampling survey on the mental health status of primary and secondary school teachers in the southeastern Chongqing region. *Journal of Chongqing University of Science and Technology*, (03), 83–85. doi:10.19406/j.cnki.cqkjxyxbskb.2005.03.024

Lin H. L. (2004). Mental health status of primary and secondary school teachers in Shaoxing City. *Chinese Journal of School Health*, (01), 90.

Lin H. L. (2005). Investigation on the mental health status of famous teachers in elementary schools and middle schools in Shaoxing City. *Chinese Journal of Tissue Engineering Research*, (12), 100–101.

Lin X. (2005). Investigation and research on the mental health status of secondary school physical education teachers in Longyan City. *Journal of Longyan University*, (03), 114–115+118. doi:10.16813/j.cnki.cn35-1286/g4.2005.03.045

Liu B., Liu J. W., Li L., Wang Y. J., & Qian Y. (2005). Analysis of SCL-90 assessment results of primary and secondary school teachers of different ethnic groups in Urumqi City. *Journal of Xinjiang Medical University*, 28(05), 414–416.

Liu D. F., & Zhang N. (2010). Analysis of mental health status and influencing factors of non-permanent primary school

teachers. *Journal of Hefei University (Social Sciences)*, 27(03), 110–113.

Liu J. M. (2010). *Research on the mental health of Southwest primary and secondary school teachers and its related factors* (Master's thesis, Southwest University).

Liu S. Q., Zhao C. P., & Liu S. W. (2004). Research on mental health status and countermeasures of primary and secondary school physical education teachers in Hebei Province. *Journal of Hebei Institute of Physical Education*, (01), 11–12.

Liu W. (2009). *Research on the mental health status of junior high school teachers in Guiyang and its countermeasures* (Master's thesis, Northeast Normal University).

Liu W. J. (2007). *The status and counterplan of the teachers' phycologial health in the HeLin League in HuHeHot* (Master's thesis, Inner Mongolia Normal University).

Liu X. W. (2007). An investigation and analysis of the psychological problems among the teachers in Qingtian middle and primary school. *Journal of Lishui University*, 29(01), 118–122.

Liu Y., & Guo C. (2012). A survey and analysis of the mental health status of primary and secondary school teachers in Southwest China. *Chinese Journal of Health Education*, 28(10), 843–845+855. doi:10.16168/j.cnki.issn.1002-9982.2012.10.009

Liu Y. H. (2007). *The research on situations of Mongolian and Han primary and middle school teachers' mental health and defense mechanisms* (Master's thesis, Inner Mongolia Normal University).

Liu Y. L. (2008). Status of mental health of junior school teachers in underdeveloped areas. *China Journal of Health Psychology*, 16(02), 221–223. doi:10.13342/j.cnki.cjhp.2008.02.051

Long Q. Z. (2006). Studies on the survey of the primary school young teachers' mental health in Tibetan Autonomous Region. *Journal of Tibet University for Nationalities (Philosophy and Social Sciences Edition)*, 27(04), 44–47+107.

Lu H. L., Yuan Y. F., Hu B., Guo M., & Chao X. L. (2011). Study on mental health status of female teachers in primary and secondary schools in economically underdeveloped areas. *Modern Preventive Medicine*, 38(14), 2776–2777+2780.

Luo X. L. (2006). An investigation on the mental health of teachers in remote mountain areas. *Chinese Journal of Clinical Rehabilitation*, 10(06), 165–167.

Luo Y., Xu Y. Y., & Liang Y. (2013). Mental health status of middle school teachers in Henan Province. *China Journal of Health Psychology*, 21(03), 387–389. doi:10.13342/j.cnki.cjhp.2013.03.010

Ma C. F. (2018). The impact of job burnout on the mental health of boarding primary and secondary school teachers in Tibetan areas of Qinghai Province. *Journal of Research on Education for Ethnic Minorities*, 29(02), 31–38. doi:10.15946/j.cnki.1001-7178.2018.02.004

Ma H. X., & Han X. M. (2004). Analysis of pre-service SCL-90 test results of mental health education teachers in key high schools. *Theory and Practice of Education*, (12), 53–55.

Pan K. Q., Xie J. L., Liu S. H., Yi Y. M., & Zhao Y. (2013). Investigation and analysis of the mental health status of 365 primary and secondary school teachers. *Medical Journal of West China*, 25(06), 931–933.

Pan X. F., & He H. Y. (2006). Correlation analysis between school interpersonal atmosphere and teachers' mental health. *Journal of Xiangnan University*, 27(06), 109–112.

Peng Y. H., Hu D. L., & Lin F. (2012). A survey and analysis of the mental health status of rural primary and secondary school teachers. *Education Research Monthly*, (02), 35–37. doi:10.16477/j.cnki.issn1674-2311.2012.02.013

Qiu L. (2003). Mental health status of primary and secondary school teachers in Guangdong Province. *Chinese Journal of Clinical Psychology*, (03), 216–218. doi:10.16128/j.cnki.1005-3611.2003.03.021

Ren Q. P. (2006). Investigation and research on the mental health status of rural primary and secondary school teachers. *Journal of Changzhou Institute of Technology (Social Science Edition)*, (03), 37–42.

She L. Z., & Liu S. J. (2009). Survey on mental health of primary school teachers in Ningyuan County. *Journal of Hunan University of Science and Engineering*, 30(07), 228–230.

Shen Z. D., Gong J. Y., Wang J., Zhang J., & Zhu Y. J. (2004). The effect of stress management training of primary school teachers. *Chinese Mental Health Journal*, 18(05), 316–317.

Shen Z. R., & Chen L. (2005). Analysis of teachers' mental health and its influencing factors. *Chinese Journal of Behavioral Medical Science*, 14(04), 353–354.

Sheng S. Z., Zhu L. H., Chen A. J., & Sun B. (2004). Investigation on mental health of middle school physical education backbone teachers in Shandong Province. *Journal of Sports Science*, (02), 54–55+65. doi:10.16469/j.css.2004.02.017

Song Z. H., & Luo X. (2011). A comparative study on the current situation of mental health and social support of teachers in eastern and western China. *Education Research Monthly*, (04),48–50. doi:10.16477/j.cnki.issn1674-2311.2011.04.009

Su B. Y., Li J. X., & Li W. (2013). Correlational research between job satisfaction and mental health of primary and secondary school teachers. *Journal of Educational Development*, (08), 30–32. doi:10.16215/j.cnki.cn44-1371/g4.2013.08.025

Sun Y. Q. (2008). Investigation and analysis on the mental health status of physical education teachers in primary and secondary schools in Jilin Province. *Journal of Jilin Institute of Physical Education*, (05), 96–97.

Sun Z. (2011). Investigation and research on mental health status of rural junior high school teachers in Zhoukou City. *Journal of Zhoukou Normal University*, 28(02), 151–153. doi:10.13450/j.cnki.jzknu.2011.02.036

Tang Z. Q., & Wang Y. (2007). A survey and strategies on the mental health condition of primary school teachers in Tianjin. *Journal of Tianjin Normal University (Elementary Education Edition)*, (03), 29–33.

Wang C., Jin C. S., Li C. Q., & Yu B. S. (2004). Analysis on the measurement with SCL-90 questionaire on middle school teachers. *Journal of Shenyang Medical College*, 6(03), 152–153.

Wang D. C. (2012). A comparative study of mental health levels of primary and middle school teachers in different school stages. *Education Exploration*, (11), 141–142.

Wang H. (2005). *Analysis and countermeasures of mental health status of rural junior high school teachers in Changping District, Beijing* (Master's thesis, Capital Normal University).

Wang J. Z., Zhao M. X., Zong J. M., & Yan S. C. (2004). Research on the current situation and countermeasures of mental health of primary and secondary school teachers in Hebei Province. *Journal of Hebei Normal University (Educational Science Edition)*, (01), 69–73. doi:10.13763/j.cnki.jhebnu.ese.2004.01.013

Wang L., & Tian X. H. (2010). Survey on mental health status of primary and secondary school teachers in ethnic regions. *Educational Research and Experiment*, (05), 85–88.

Wang L. J. (2005). Analysis of teachers' mental health problems in a changing society. *Journal of Henan University (Medical Science)*, (02), 75–76. doi:10.15991/j.cnki.41-1361/r.2005.02.047

Wang L. J. (2005). On orthopsychiatry of secondary school and elementary school teachers. *Henan Social Sciences*, (03), 138–141.

Wang M. H., & Li L. (2004). The investigation on the growth phase's psychological health situation of new teachers from primary schools in Sifang District of Qingdao. *Journal of Qingdao Technical College*, 17(03), 53–56.

Wang W. Y., Chen Z. Q., Huo Y., & Zhou Y. H. (2010). Investigation and countermeasure research on mental health status of primary and secondary school teachers. *Journal of Jiangsu Institute of Education (Social Science)*, 26(03), 37–39.

Wang W. Z., & Guo L. Y. (2007). An investigation on occupational stress, teacher burnout and mental health state of primary and middle schools' teachers and the relations among them. *Chinese Journal of Clinical Psychology*, (02), 146–148. doi:10.16128/j.cnki.1005-3611.2007.02.013

Wang X. M., Meng W. J., & Han X. Y. (2012). A study on the mental health status of bilingual teachers of Uyghur ethnicity in primary and secondary schools and its countermeasures. *Contemporary Education and Culture*, 4(05), 27–34. doi:10.13749/j.cnki.cn62-1202/g4.2012.05.008

Wang X. Q., Hu G. J., Wu H. Q., & Wang H. R. (2004). Investigation and analysis on mental health status of key middle school teachers. *China Journal of Health Psychology*, 12(02), 126–128. doi:10.13342/j.cnki.cjhp.2004.02.018

Wang Y. C., & Wang H. M. (2014). Study on mental health status of primary and secondary school teachers in some regions of Jilin Province. *Staff and Worker's Education of China*, (22), 67–68.

Wang Y. M., Wei C. J., Chen K. D., Ren X. W., Li J., Wang Y., & Zhang Y. Q. (2014). Investigation of mental health status among 223 middle school teachers in Lanzhou. *Modern Preventive Medicine*, 41(20), 3728–3730+3737.

Wang Y. Z. (2000). Investigation on mental health of young teachers in rural primary and secondary schools. *Youth Studies*, (09), 23–26.

Wang Z. C. (2013). *A study on the relationships among occupational stress, coping style, social support and mental health status of high school teachers* (Master's thesis, Inner Mongolia Normal University).

Wang Z. C., Zhao Y L. (2008). Investigation and Analysis of Psychologically Healthy Condition of the Yuanmou County Grade Teacher. *Journal of Qujing Normal University*, 2008 (03): 89-92.

Wang Z. M., & Shao Y. J. (2006). A comparative study of the mental health of principal and regular high school teachers. *Journal of Hebei Normal University (Educational Science Edition)*, (04), 72–78. doi:10.13763/j.cnki.jhebnu.ese.2006.04.012

Wei K. X., & Li J. (2006). The investigation and analysis of the condition of mental health in middle school teachers in 2003. *Preventive Medicine Tribune*, (01), 27–28.

Wei Q. (2009). A survey of private primary school teachers' mental health. *Journal of Chinese Culture*, (02), 174–177.

Wei Y. Y., & Xiong M. (2008). Correlation study on occupational stress and mental health of primary and secondary school teachers. *Journal of Psychiatry*, 21(04), 268–270.

Wu A. H. (2005). *The study of primary and secondary school teachers in Shanghai, behavioral and mental health status* (Master's thesis, Shanghai Normal University).

Wu B. R. (2014). *Investigation and analysis of the mental health status of middle school teachers in the new era* (Master's thesis, Hunan Normal University).

Wu H. Y. (2014). Investigation and analysis of mental health status of middle school teachers in Neijiang City. *Modern Preventive Medicine*, 41(15), 2720–2723.

Wu X. M. (2018). *A study on the impact of work stress and personality traits on the psychosomatic health of primary and secondary school teachers* (Master's thesis, Shanghai Normal University).

Wu Z. X. (2002). Investigation on mental health of middle school teachers. *Education Exploration*, (05), 76–78.

Xiang Z. Q., & Xing H. (2004). Investigation and research on the current mental health status of primary and secondary school teachers in Guangzhou. *Journal of Guangzhou University (Social Science Edition)*, (09), 87–91+96.

Xiao S. B., & Zhong S. Y. (2005). The state of psychological soundness of primary school teachers in Hainan. *Journal of Hainan Normal University (Social Sciences Edition)*, (06), 148–150. doi:10.16061/j.cnki.cn46-1076/c.2005.06.036

Xiao S. R., & Yang N. C. (2005). Relationship between suicidal ideation and depression and mental health among rural primary and secondary school teachers. *Chinese Journal of Clinical Psychology*, (04), 81–82. doi:10.16128/j.cnki.1005-3611.2005.04.028

Xing Q. (2010). Relationship between mental health level and self-efficacy of primary and secondary school teachers in Guangzhou. *Journal of Educational Development*, (01), 32–36. doi:10.16215/j.cnki.cn44-1371/g4.2010.01.018

Xu H. (2000). Mental health of young teachers in primary schools. *China Journal of Health Psychology*, (06), 647–649. doi:10.13342/j.cnki.cjhp.2000.06.022

Yang H. B., Fang M., & Cao J. X. (2015). An analysis of mental health status of bilingual primary school mathematics teachers in border areas — A case study of a city in Kizilsu Kirghiz Autonomous Prefecture, Xinjiang. *Journal of Beijing Institute of Education (Natural Science Edition)*, 10(04), 22–26+32. doi:10.16398/j.cnki.jbjie(n)issn1673-6923.2015.04.005

Yang L. J., Xu Y., & Chen K. (2006). Analysis on mental health status of 520 primary and middle school teachers in Suzhou area. *Chinese Journal of School Health*, (08), 698–699.

Yang S. Y., Huo Y. S., Zong Y. S., Mu Z. Q., Guo M. F., Kang H. Y., Li C. L., & Yang J. K. (2003). Mental health of rural primary and secondary school teachers in Yuanjiang County. *China Journal of Health Psychology*, (04), 309–311. doi:10.13342/j.cnki.cjhp.2003.04.029

Yang Y., & Xu Y. (2019). A survey on the current status of primary school teachers' mental health level in southern Jiangsu. *Comparative Study of Cultural Innovation*, 3(32), 29–30.

Yang Y. S., Zuo X., & Liang X. L. (2007). Investigation and research on the mental health status of primary school teachers in Inner Mongolia. *Journal of Inner Mongolia Normal University (Natural Science Edition)*, (02), 172–177.

Ye F. H., Wang B. H., Jia J. P., Zhao M. Q., Yao J., Feng N., …, & Na L. (2011). Investigation on mental health status of primary and secondary school teachers in Taiyuan City, Shanxi Province. *Chinese Journal of Health Education*, 27(05), 398–399. doi:10.16168/j.cnki.issn.1002-9982.2011.05.021

Yu D. J., Li L. L., & Ye L. J. (2008). Investigation and analysis on the mental health of middle school teachers in Shenyang. *Modern Preventive Medicine*, (18), 3572–3573.

Yu H. T. (2013). *A study on the mental health problems of primary and secondary school teachers and its countermeasures* (Master's thesis, Northeast Normal University).

Yu J. (2004). *A study on mental health status of middle school teachers and its influencing factors* (Master's thesis, Northeast Normal University).

Yu Q. M., Li L., Zhou B., & Liao J. (2015). Investigation and analysis of mental health status of primary and secondary school teachers in Yunnan Province. *China Journal of Health Psychology*, 23(02), 201–204. doi:10.13342/j.cnki.cjhp.2015.02.014

Yu X. X. (2003). Investigation on mental health of key teachers in primary and secondary schools. *Education Exploration*, (06), 80–82.

Yue C. Z., Luo W. W., Pan W. G., Yang Z. W., & Lin X. T. (2021). Analysis of mental health status and influencing factors of primary and secondary school teachers in Chongqing. *Health Medicine Research and Practice*, 18(04), 45–50.

Zhang B., Wang M., & Jiang Y. G. (2022). A survey report on the mental health of primary and secondary school teachers in a new-era county — A case study of Guanghan City. *Education Science Forum*, (17), 11–14.

Zhang D. X. (2003). Study on mental health of primary and secondary school teachers. *Journal of Binzhou Normal University*, (04), 76–78.

Zhang L., Zhao J., Xiao H., Zheng H., Xiao Y., Chen M., & Chen D. (2014). Mental health and burnout in primary and

secondary school teachers in the remote mountain areas of Guangdong Province in the People's Republic of China. *Neuropsychiatric Disease and Treatment*, 2014: 123–130.

Zhang L. P. (2010). An investigation on current mental health status of 78 rural junior high school key teachers. *Journal of Anyang Normal University*, (06), 125–129. doi:10.16140/j.cnki.1671-5330.2010.06.027

Zhang L. L., Xiao H. Q., Zheng H. B., Xiao Y. N., Chen M. Y., Chen D. L., …, & Zhao Z. H. (2013). Investigation on mental health of primary and secondary school graduating class teachers in a city. *China Journal of Health Psychology*, 21(07), 1059–1061. doi:10.13342/j.cnki.cjhp.2013.07.010

Zhang P., & Dong J. W. (2015). Investigation and analysis of mental health status of rural primary and secondary school teachers in Southern Xinjiang. *Journal of Xinjiang Education Institute*, 31(04), 42–45.

Zhang Q. (2002). Survey on mental health of primary school teachers. *Journal of Sichuan Normal University (Social Sciences Edition)*, (04), 130–133. doi:10.13734/j.cnki.1000-5315.2002.04.025

Zhang S. F., Ma C. S., & Meng G. (2003). Analysis of SCL-90 among junior middle school teachers in Zhengzhou area. *Medical Journal of Chinese People's Health*, (05), 319–320.

Zhang W., & Deng B. (2017). Mental health status of psychological teachers in primary and secondary schools in Guizhou Province. *Occupation and Health*, 33(05), 589–592. doi:10.13329/j.cnki.zyyjk.2017.0179

Zhang W. F. (2006). Research on the mental health level of physical education teachers. *Education and Vocation*, (33), 179–180. doi:10.13615/j.cnki.1004-3985.2006.33.027

Zhang X. (2009). Investigation and research on the mental health status of primary and secondary school teachers in ethnic areas of Guizhou Province. *Journal of Southwest Guizhou Teachers College for Nationalities*, (01), 67–70.

Zhang X. Q., Fan C. L., Lin Z., Chen D. Q., Lu H. Y., Wang W., Zhou J.B., & Lin S. K. (2015). Investigation on mental health status of teachers in Li and Miao ethnic mountainous areas. *China Journal of Health Psychology*, 23(11), 1658–1661. doi:10.13342/j.cnki.cjhp.2015.11.017

Zhang X. X. (2016). Investigation and research on the current mental health status of primary and secondary school teachers in Linwei District, Weinan City. *Western China Quality Education*, 2(03),143–144. doi:10.16681/j.cnki.wcqe.201603106

Zhang Y. F. (2005). An investigation and study on mental health of primary and secondary school teachers. *Journal of Shanxi Radio & TV University*, (04), 48–49.

Zhang Y. Q. (2010). *A survey of the current situation of primary and secondary school teachers' mental health in Qu County and suggestions for its maintenance* (Master's thesis, Sichuan Normal University).

Zhang Y. Z., Deng X. L., & Li W. H. (2006). Investigation of mental health state of junior high school teachers in underdevelopment area in country. *China Journal of Health Psychology*, (01), 83–85. doi:10.13342/j.cnki.cjhp.2006.01.033

Zhao H. L. (2011). The survey of elementary and middle school teachers' mental health status in rural areas. *Journal of Pingdingshan University*, 26(01), 122–125.

Zhao M. Y. (2007). Assessment of quality of life and mental health in teachers from different type schools. *Journal of Clinical Rehabilitative Tissue Engineering Research*, 11(17), 3266–3269.

Zheng Y. J., Yin X. Y., & Long J. (2015). Prediction of mental health level by job burnout and coping styles among primary school teachers. *China Journal of Health Psychology*, 23(11), 1646–1651. doi:10.13342/j.cnki.cjhp.2015.11.014

Zheng Y. Z. (2007). Current situation of mental health of middle school physical education teachers and the training objectives of quality education in college physical education. *Journal of Shaoxing University (Natural Science Edition)*, (10), 98–100.

Zhou C. E., Zhang X. X., & Hong R. Y. (2006). Investigation and analysis on the mental health of female teachers in primary

and secondary schools. *Teacher Education Research*, (01), 71–75. doi:10.13445/j.cnki.t.e.r.2006.01.015

Zhou H. M. (2015). Effect of social support to mental health: The intermediary role of self-efficacy — Taking primary and secondary school teachers in Heze as an example. *Journal of Heze University*, 37(02), 93–96. doi:10.16zhao/j.cnki.37-1436/z.2015.02.023

Zhou L., Meng H. Q., Qiu H. T., & Luo Q. H. (2008). An investigation and analysis on the mental health status of rural teachers in Chongqing. *Chongqing Medicine*, (13), 1475–1477.

Zhou X. Q., Li X. S., Wang L., Xia H. S., Cai C. L., Liu H., …, & Wu Q. (2006). Investigation and study on mental health level of primary school teachers in Hefei City. *China Journal of Health Psychology*, (01), 99–101. doi:10.13342/j.cnki.cjhp.2006.01.040

Zhou Y. X. (2008). *Research on teacher's psychological health in middle and primary school* (Master's thesis, Central China Normal University).

Zhu X. L., & Zhang C. Y. (2000). Analysis on mental health of teachers in the middle and primary schools in the country areas in Tongcheng. *Chinese Journal of Disease Control and Prevention*, (02), 154–155.

Supplementary file 3. Assessment of risk of bias

Study name	I1	I2	I3	I4	I5	I6	I7	I8	Overall Score
Zhu, X. L. et al. (2000)	Y	Y	Y	Y	Y	Y	Y	Y	8
Wang, Y. Z. (2000)	Y	Y	Y	Y	Y	Y	Y	Y	8
Xu, H. (2000)	Y	Y	Y	Y	Y	Y	Y	Y	8
Xiao, S. R. et al. (2005)	Y	Y	Y	Y	Y	Y	Y	Y	8
Wu, Z. X. (2002)	Y	Y	Y	Y	N	N	Y	N	5
Li, C. L. (2002)	Y	Y	Y	Y	Y	Y	Y	Y	8
Chen, J. F. et al. (2002)	Y	Y	Y	Y	Y	Y	N	Y	7
Zhang, Q. (2002)	Y	Y	Y	Y	Y	Y	Y	Y	8
Dong, W. J. (2002)	Y	Y	Y	Y	Y	Y	Y	Y	8
Yu, X. X. (2003)	Y	Y	Y	Y	N	N	Y	N	5
Yang, S. Y. et al.(2003)	Y	Y	Y	Y	Y	Y	Y	Y	8
Zhang, D. X. (2003)	Y	Y	Y	Y	N	Y	Y	Y	7
Qiu, L. (2003)	Y	Y	Y	Y	N	Y	Y	Y	7
Zhang, S. F. et al. (2003)	Y	Y	Y	Y	N	Y	Y	Y	7
Li, J., Liu et al. (2003)	Y	Y	Y	Y	N	N	Y	N	5
Sheng, S. Z. et al. (2004)	Y	Y	Y	Y	N	N	N	N	4
Feng, W. et al. (2004)	Y	Y	Y	Y	N	N	Y	N	5
Li, J. Q. (2004)	Y	Y	Y	Y	Y	Y	Y	Y	8
Ding, F. Q. (2004)	Y	Y	Y	Y	Y	Y	Y	Y	8
Shen, Z. R. et al. (2005)	Y	Y	Y	Y	N	N	Y	N	5
Wang, C. et al. (2004)	Y	Y	Y	Y	N	N	Y	N	5
Shen, Z. D. et al. (2004)	Y	Y	Y	Y	N	N	N	N	4
Wang, X. Q. et al. (2004)	Y	Y	Y	Y	N	N	Y	N	5
Li, B. Z. et al. (2004)	Y	Y	Y	Y	N	Y	Y	Y	7
Yu, J. (2004)	Y	Y	Y	Y	N	N	Y	N	5
Li, J. (2004)	Y	Y	Y	Y	N	Y	Y	Y	7
Li, F. Q. (2004)	Y	Y	Y	Y	N	Y	Y	Y	7
Xiang, Z. Q. et al. (2004)	Y	Y	Y	Y	Y	Y	Y	Y	8
Liu, S. Q. et al. (2004)	Y	Y	Y	Y	N	N	Y	N	5
Wang, J. Z. et al. (2004)	Y	Y	Y	Y	Y	Y	Y	Y	8
Lin, H. L. (2004)	Y	Y	Y	Y	N	Y	Y	Y	7
Wang, M. H. et al. (2004)	Y	Y	Y	Y	Y	Y	Y	Y	8
Chen, Q. Y. et al. (2004)	Y	Y	Y	Y	Y	Y	Y	Y	8
Li, J. et al (2003)	Y	Y	Y	Y	Y	Y	Y	Y	8
Liu, B. et al. (2005)	Y	Y	Y	Y	Y	Y	Y	Y	8
Wang, L. J. (2005)	Y	Y	Y	Y	N	Y	Y	Y	7
Wang, L. J. (2005)	Y	Y	Y	Y	Y	Y	Y	Y	8
Zhang, Y. F. (2005)	Y	Y	Y	Y	N	Y	Y	Y	7

Wu, A. H. (2005)	Y	Y	Y	Y	Y	Y	Y	Y	8
Cheng, M. (2005)	Y	Y	Y	Y	Y	Y	Y	Y	8
Liao, Q. M. et al. (2005)	Y	Y	Y	Y	Y	Y	Y	Y	8
Xiao, S. B. et al. (2005)	Y	Y	Y	Y	N	Y	Y	Y	7
Lin, X. (2005)	Y	Y	Y	Y	N	Y	Y	Y	7
Ma, H. X. et al. (2004)	Y	Y	Y	Y	N	N	N	N	4
Zhou, C. E. et al. (2006)	Y	Y	Y	Y	N	Y	Y	Y	7
Ren, Q. P. (2006)	Y	Y	Y	Y	Y	Y	Y	Y	8
Chen, S. (2006)	Y	Y	Y	Y	Y	Y	Y	Y	8
Cai, Y. Q. (2006)	Y	Y	Y	Y	Y	Y	Y	Y	8
Deng, W. G. et al. (2006)	Y	Y	Y	Y	N	Y	Y	Y	7
Pan, X. F. et al (2006)	Y	Y	Y	Y	N	Y	Y	Y	7
Wang, Z. M.	Y	Y	Y	Y	N	N	Y	N	5
Zhang, Y. Z. et al. (2006)	Y	Y	Y	Y	Y	Y	Y	Y	8
Zhou, X. Q. et al. (2006)	Y	Y	Y	Y	Y	Y	Y	Y	8
Gui, Q. R. (2006)	Y	Y	Y	Y	Y	Y	Y	Y	8
Fang, H. L. et al. (2007)	Y	Y	Y	Y	N	N	Y	N	5
Yang, L. J. et al. (2006)	Y	Y	Y	Y	N	Y	Y	Y	7
Lin, H. L. (2005)	Y	Y	Y	Y	Y	Y	N	Y	7
Long, Q. Z. (2006)	Y	Y	Y	Y	N	Y	Y	Y	7
Zhang, W. F. (2006)	Y	Y	Y	Y	Y	Y	Y	Y	8
Luo, X. L. (2006)	Y	Y	Y	Y	Y	Y	Y	Y	8
Wei, K. X. et al. (2006)	Y	Y	Y	Y	N	Y	Y	Y	7
Wang, W. Z. et al. (2007)	Y	Y	Y	Y	N	N	Y	N	5
Du, L. J. (2007)	Y	Y	Y	Y	N	Y	Y	Y	7
Zheng, Y. Z. (2007)	Y	Y	Y	Y	Y	Y	Y	Y	8
Dong, A. G. et al. (2007)	Y	Y	Y	Y	Y	Y	Y	Y	8
Ding, Y. L. (2007)	Y	Y	Y	Y	Y	Y	Y	Y	8
Li, M. Y. (2007)	Y	Y	Y	Y	N	Y	Y	Y	7
Wang, H. (2005)	Y	Y	Y	Y	Y	Y	Y	Y	8
Tang, Z. Q. et al. (2007)	Y	Y	Y	Y	Y	Y	Y	Y	8
Yang, Y. S. et al. (2007)	Y	Y	Y	Y	N	N	Y	N	5
Chen, X. Y. et al. (2007)	Y	Y	Y	Y	N	N	Y	N	5
Liu, X. W. (2007)	Y	Y	Y	Y	Y	Y	Y	Y	8
Zhou, L. et al. (2008)	Y	Y	Y	Y	Y	Y	Y	Y	8
Wei, Y. Y. et al. (2008)	Y	Y	Y	Y	Y	Y	Y	Y	8
Sun, Y. Q. (2008)	Y	Y	Y	Y	N	Y	Y	Y	7
Ju, X. M. (2008)	Y	Y	Y	Y	Y	Y	Y	Y	8
Liu, Y. H. (2007)	Y	Y	Y	Y	Y	Y	Y	Y	8
Liu, W. J. (2007)	Y	Y	Y	Y	Y	Y	Y	Y	8
Zhou, Y. X. (2008)	Y	Y	Y	Y	Y	Y	Y	Y	8

Yu, D. J. et al. (2008)	Y	Y	Y	Y	Y	Y	Y	Y	8
Li, F. Y. et al. (2008)	Y	Y	Y	Y	N	N	Y	N	5
Wei, Q. (2009)	Y	Y	Y	Y	N	N	N	N	4
Liu, Y. L. (2008)	Y	Y	Y	Y	N	Y	Y	Y	7
Zhao, M. Y. (2007)	Y	Y	Y	Y	Y	Y	Y	Y	8
Wang. Z. C. et al. (2008)	Y	Y	Y	Y	Y	Y	Y	Y	8
Li, F. Y. et al. (2008)	Y	Y	Y	Y	Y	Y	Y	Y	8
Zhang, X. (2009)	Y	Y	Y	Y	Y	Y	Y	Y	8
Huang, X. Y. et al. (2009)	Y	Y	Y	Y	Y	Y	Y	Y	8
She, L. Z. et al. (2009)	Y	Y	Y	Y	Y	Y	Y	Y	8
Chen, D. R. (2009)	Y	Y	Y	Y	Y	Y	Y	Y	8
Hu, M. H. (2007)	Y	Y	Y	Y	Y	Y	Y	Y	8
Liu, W. (2009)	Y	Y	Y	Y	Y	Y	Y	Y	8
Huang, X. Y. et al. (2009)	Y	Y	Y	Y	N	N	Y	N	5
Li, C. J. (2008)	Y	Y	Y	Y	N	Y	Y	Y	7
Xing, Q. (2010)	Y	Y	Y	Y	Y	Y	Y	Y	8
He, S. D. (2010)	Y	Y	Y	Y	Y	Y	Y	Y	8
Hu, W. P. et al. (2010)	Y	Y	Y	Y	Y	Y	Y	Y	8
Wang, L. et al. (2010)	Y	Y	Y	Y	Y	Y	N	Y	7
Liu, D. F. et al. (2010)	Y	Y	Y	Y	Y	Y	N	Y	7
Wang, W. Y. et al. (2010)	Y	Y	Y	Y	N	Y	Y	Y	7
Liu, J. M. (2010)	Y	Y	Y	Y	N	N	Y	N	5
Li, J. P. (2010)	Y	Y	Y	Y	N	Y	Y	Y	7
Ye, F. H. et al. (2011)	Y	Y	Y	Y	Y	Y	Y	Y	8
Lu, H. L. et al. (2011)	Y	Y	Y	Y	Y	Y	Y	Y	8
Zhang, Y. Q. (2010)	Y	Y	Y	Y	Y	Y	Y	Y	8
Chen, L. J. et al. (2011)	Y	Y	Y	Y	Y	Y	Y	Y	8
Zhao, H. L. (2011)	Y	Y	Y	Y	Y	Y	Y	Y	8
Sun, Z. (2011)	Y	Y	Y	Y	Y	Y	Y	Y	8
Song, Z. H. et al. (2011)	Y	Y	Y	Y	N	N	Y	N	5
Zhang, L. P. (2010)	Y	Y	Y	Y	Y	Y	N	Y	7
Wang, D. C. (2012)	Y	Y	Y	Y	Y	Y	Y	Y	8
Peng, Y. H. et al. (2012)	Y	Y	Y	Y	Y	Y	Y	Y	8
Wang, X. M. et al. (2012)	Y	Y	Y	Y	Y	Y	Y	Y	8
Liu, Y. et al. (2012)	Y	Y	Y	Y	N	N	Y	N	5
Luo, Y. et al. (2013)	Y	Y	Y	Y	N	Y	Y	Y	7
Hu, X. et al. (2012)	Y	Y	Y	Y	N	N	Y	N	5
Gao, M. S. (2011)	Y	Y	Y	Y	N	N	Y	N	5
Pan, K. Q. et al. (2013)	Y	Y	Y	Y	Y	Y	Y	Y	8
Yu, H. T. (2013)	Y	Y	Y	Y	Y	Y	Y	Y	8
Wang, Z. C. (2013)	Y	Y	Y	Y	Y	Y	Y	Y	8

Chen, Z. M. (2013)	Y	Y	Y	Y	N	Y	Y	Y	7
Zhang, L. L. et al. (2013)	Y	Y	Y	Y	Y	Y	Y	Y	8
Su, B. Y. et al. (2013)	Y	Y	Y	Y	Y	Y	Y	Y	8
Hu, H. Y. et al. (2014)	Y	Y	Y	Y	Y	Y	Y	Y	8
Han, M. M. et al. (2014)	Y	Y	Y	Y	Y	Y	Y	Y	8
Wu, B. R. (2014)	Y	Y	Y	Y	Y	Y	Y	Y	8
Wang, Y. M. et al. (2014)	Y	Y	Y	Y	Y	Y	Y	Y	8
Wu, H. Y. (2014)	Y	Y	Y	Y	Y	Y	Y	Y	8
Zhang, L. et al. (2014)	Y	Y	Y	Y	Y	Y	Y	Y	8
Wang, Y. C. et al. (2014)	Y	Y	Y	Y	N	N	Y	N	5
Yu, Q. M. et al. (2015)	Y	Y	Y	Y	N	Y	Y	Y	7
Chen, Z. L. ET AL. (2015)	Y	Y	Y	Y	Y	Y	Y	Y	8
Zhang, P. et al. (2015)	Y	Y	Y	Y	Y	Y	Y	Y	8
Yang, H. B. et al. (2015)	Y	Y	Y	Y	Y	Y	Y	Y	8
Zheng, Y. J. et al. (2015)	Y	Y	Y	Y	Y	Y	Y	Y	8
Kang, Y. W. et al. (2015)	Y	Y	Y	Y	N	Y	Y	Y	7
Zhou, H. M. (2015)	Y	Y	Y	Y	Y	Y	Y	Y	8
Zhang, X. Q. et al. (2015)	Y	Y	Y	Y	Y	Y	N	Y	7
Guan, T. et al. (2015)	Y	Y	Y	Y	Y	Y	Y	Y	8
Fan, X. Y. (2016)	Y	Y	Y	Y	Y	Y	Y	Y	8
Zhang, X. X. (2016)	Y	Y	Y	Y	Y	Y	Y	Y	8
Zhang, W. et al. (2017)	Y	Y	Y	Y	N	Y	Y	Y	7
Ma, C. F. (2018)	Y	Y	Y	Y	Y	Y	Y	Y	8
Wu, X. M. (2018)	Y	Y	Y	Y	Y	Y	Y	Y	8
Li, X. (2018)	Y	Y	Y	Y	N	N	Y	N	5
Dong, Y. X. (2019)	Y	Y	Y	Y	Y	Y	Y	Y	8
Deng, X. B. et al. (2020)	Y	Y	Y	Y	Y	Y	Y	Y	8
Yang, Y. et al. (2019)	Y	Y	Y	Y	Y	Y	Y	Y	8
Yue, C. Z. et al. (2021)	Y	Y	Y	Y	Y	Y	Y	Y	8
Gao, T. L. (2021)	Y	Y	Y	Y	Y	Y	Y	Y	8
Zhang, B. et al. (2022)	Y	Y	Y	Y	Y	Y	Y	Y	8
Jiang, Y. et al. (2022)	Y	Y	Y	Y	Y	Y	Y	Y	8
Fang, G. X. et al. (2023)	Y	Y	Y	Y	Y	Y	Y	Y	8

Note: high quality, ≥ 6 points, low risk of bias; moderate quality, 4~5 points, a certain degree of bias, low quality, ≤3 points, relatively high risk of bias.

Supplementary file 4. Forest plot

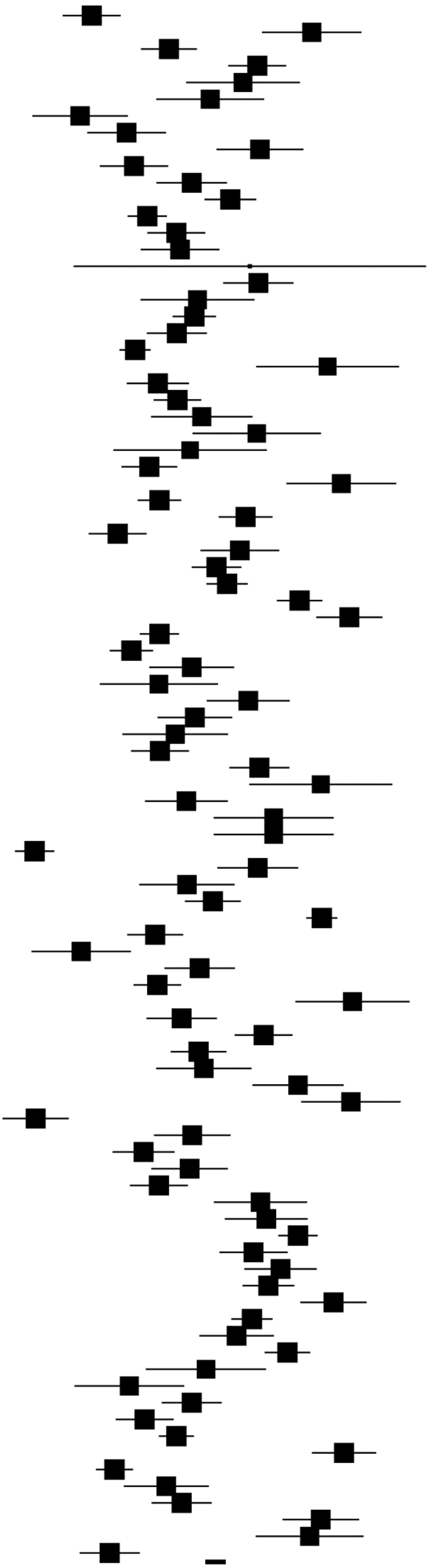

MD	95%CI	Weight
-0.06	[-0.11, -0.00]	0.67 %
0.36	[0.27, 0.46]	0.64 %
0.09	[0.04, 0.14]	0.67 %
0.26	[0.20, 0.31]	0.67 %
0.23	[0.12, 0.34]	0.62 %
0.17	[0.07, 0.27]	0.63 %
-0.08	[-0.17, 0.01]	0.64 %
0.01	[-0.06, 0.09]	0.66 %
0.26	[0.18, 0.35]	0.65 %
0.02	[-0.04, 0.09]	0.66 %
0.13	[0.07, 0.20]	0.66 %
0.21	[0.16, 0.26]	0.68 %
0.05	[0.01, 0.09]	0.68 %
0.10	[0.05, 0.16]	0.67 %
0.11	[0.04, 0.19]	0.66 %
0.24	[-0.09, 0.58]	0.33 %
0.26	[0.19, 0.33]	0.66 %
0.14	[0.04, 0.25]	0.62 %
0.14	[0.10, 0.18]	0.68 %
0.11	[0.05, 0.16]	0.67 %
0.03	[-0.00, 0.06]	0.69 %
0.39	[0.26, 0.53]	0.59 %
0.07	[0.01, 0.13]	0.67 %
0.11	[0.06, 0.15]	0.68 %
0.15	[0.06, 0.25]	0.63 %
0.26	[0.14, 0.38]	0.60 %
0.13	[-0.01, 0.28]	0.57 %
0.05	[0.00, 0.11]	0.67 %
0.42	[0.31, 0.52]	0.62 %
0.07	[0.03, 0.11]	0.68 %
0.24	[0.19, 0.29]	0.67 %
-0.01	[-0.06, 0.05]	0.67 %
0.22	[0.15, 0.30]	0.66 %
0.18	[0.13, 0.23]	0.68 %
0.20	[0.16, 0.24]	0.68 %
0.34	[0.29, 0.38]	0.68 %
0.43	[0.37, 0.49]	0.67 %
0.07	[0.04, 0.11]	0.68 %
0.02	[-0.02, 0.06]	0.68 %
0.13	[0.05, 0.21]	0.65 %
0.07	[-0.04, 0.18]	0.62 %
0.24	[0.16, 0.32]	0.65 %
0.14	[0.07, 0.21]	0.66 %
0.10	[0.00, 0.20]	0.63 %
0.07	[0.02, 0.13]	0.67 %
0.26	[0.21, 0.32]	0.67 %
0.38	[0.24, 0.51]	0.59 %
0.12	[0.05, 0.20]	0.65 %
0.29	[0.18, 0.40]	0.61 %
0.29	[0.18, 0.40]	0.61 %
-0.16	[-0.20, -0.13]	0.68 %
0.26	[0.18, 0.34]	0.65 %
0.12	[0.03, 0.22]	0.64 %
0.17	[0.12, 0.23]	0.67 %
0.38	[0.35, 0.41]	0.69 %
0.06	[0.01, 0.12]	0.67 %
-0.07	[-0.17, 0.02]	0.64 %
0.15	[0.08, 0.22]	0.66 %
0.07	[0.02, 0.11]	0.68 %
0.44	[0.33, 0.55]	0.62 %
0.12	[0.05, 0.18]	0.66 %
0.27	[0.22, 0.32]	0.67 %
0.15	[0.09, 0.20]	0.67 %
0.16	[0.07, 0.25]	0.64 %
0.34	[0.25, 0.42]	0.64 %
0.43	[0.34, 0.53]	0.64 %
-0.16	[-0.22, -0.10]	0.67 %
0.14	[0.06, 0.21]	0.66 %
0.04	[-0.02, 0.10]	0.67 %
0.13	[0.06, 0.20]	0.66 %
0.07	[0.02, 0.13]	0.67 %
0.26	[0.18, 0.35]	0.64 %
0.28	[0.20, 0.35]	0.65 %
0.34	[0.30, 0.37]	0.68 %
0.25	[0.19, 0.32]	0.66 %
0.30	[0.23, 0.37]	0.66 %
0.28	[0.23, 0.33]	0.68 %
0.40	[0.34, 0.46]	0.67 %
0.25	[0.21, 0.29]	0.68 %
0.22	[0.15, 0.29]	0.66 %
0.32	[0.27, 0.36]	0.68 %
0.16	[0.05, 0.27]	0.61 %
0.02	[-0.09, 0.12]	0.62 %
0.13	[0.08, 0.19]	0.67 %
0.04	[-0.01, 0.10]	0.67 %
0.10	[0.07, 0.14]	0.68 %
0.42	[0.36, 0.48]	0.67 %
-0.01	[-0.05, 0.02]	0.68 %
0.09	[0.01, 0.17]	0.65 %
0.12	[0.06, 0.17]	0.67 %
0.38	[0.31, 0.45]	0.66 %
0.36	[0.26, 0.46]	0.63 %
-0.02	[-0.08, 0.04]	0.67 %

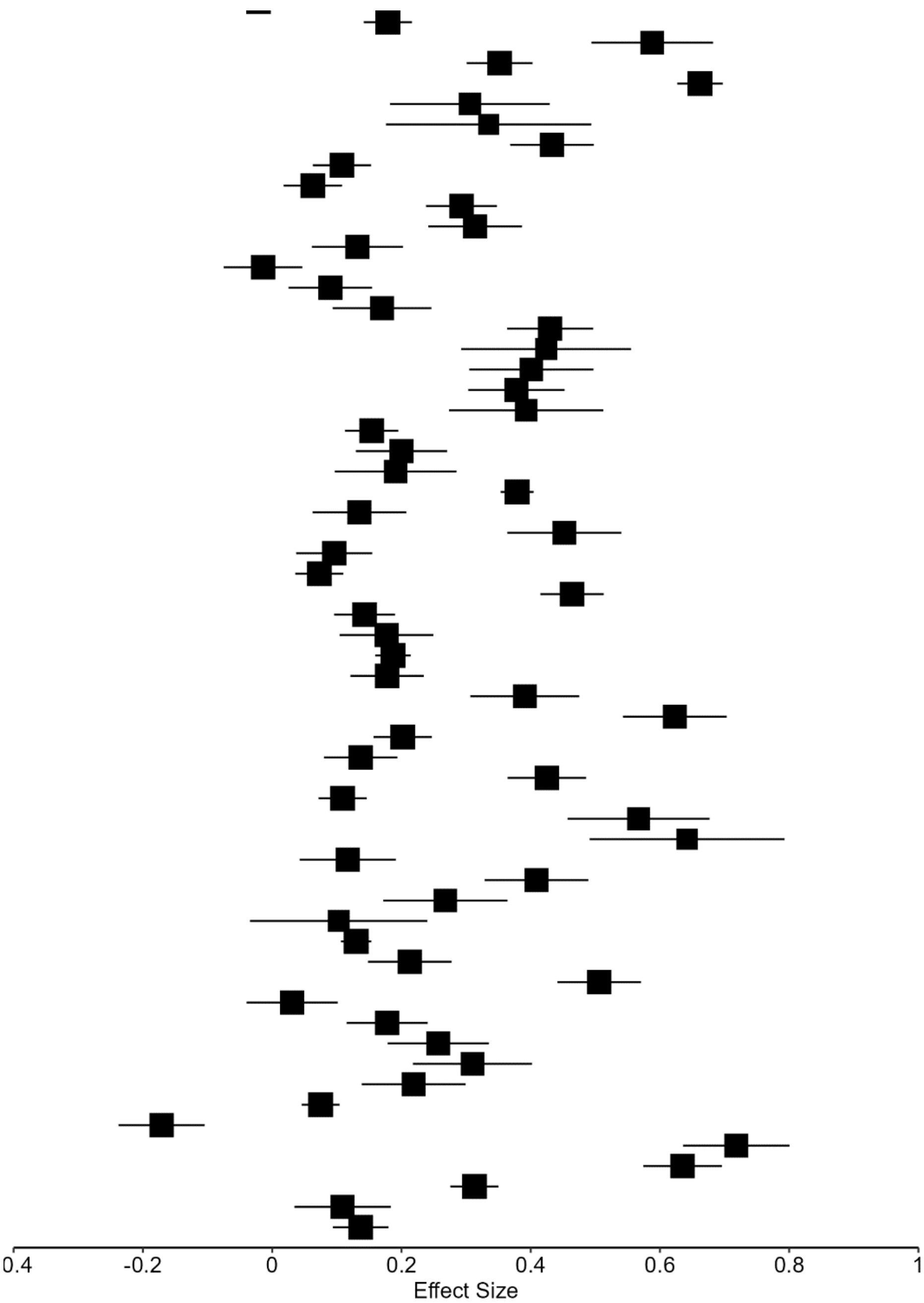

MD	95%CI	Weight
[illegible]	[illegible]	[illegible]
0.18	[0.14, 0.22]	0.68 %
0.59	[0.49, 0.68]	0.64 %
0.35	[0.30, 0.40]	0.67 %
0.66	[0.63, 0.70]	0.68 %
0.31	[0.18, 0.43]	0.60 %
0.34	[0.18, 0.49]	0.55 %
0.43	[0.37, 0.50]	0.66 %
0.11	[0.06, 0.15]	0.68 %
0.06	[0.02, 0.11]	0.68 %
0.29	[0.24, 0.35]	0.67 %
0.31	[0.24, 0.39]	0.66 %
0.13	[0.06, 0.20]	0.66 %
-0.01	[-0.07, 0.05]	0.67 %
0.09	[0.03, 0.15]	0.66 %
0.17	[0.09, 0.25]	0.65 %
0.43	[0.36, 0.50]	0.66 %
0.42	[0.29, 0.56]	0.59 %
0.40	[0.30, 0.50]	0.63 %
0.38	[0.30, 0.45]	0.66 %
0.39	[0.27, 0.51]	0.61 %
0.15	[0.11, 0.20]	0.68 %
0.20	[0.13, 0.27]	0.66 %
0.19	[0.10, 0.29]	0.64 %
0.38	[0.35, 0.40]	0.69 %
0.14	[0.06, 0.21]	0.66 %
0.45	[0.36, 0.54]	0.64 %
0.10	[0.04, 0.15]	0.67 %
0.07	[0.04, 0.11]	0.68 %
0.46	[0.42, 0.51]	0.68 %
0.14	[0.10, 0.19]	0.68 %
0.18	[0.10, 0.25]	0.66 %
0.19	[0.16, 0.21]	0.69 %
0.18	[0.12, 0.23]	0.67 %
0.39	[0.31, 0.48]	0.65 %
0.62	[0.54, 0.70]	0.65 %
0.20	[0.16, 0.25]	0.68 %
0.14	[0.08, 0.19]	0.67 %
0.42	[0.36, 0.49]	0.67 %
0.11	[0.07, 0.15]	0.68 %
0.57	[0.46, 0.68]	0.62 %
0.64	[0.49, 0.79]	0.56 %
0.12	[0.04, 0.19]	0.66 %
0.41	[0.33, 0.49]	0.65 %
0.27	[0.17, 0.36]	0.63 %
0.10	[-0.03, 0.24]	0.58 %
0.13	[0.11, 0.15]	0.69 %
0.21	[0.15, 0.28]	0.66 %
0.51	[0.44, 0.57]	0.66 %
0.03	[-0.04, 0.10]	0.66 %
0.18	[0.12, 0.24]	0.67 %
0.26	[0.18, 0.34]	0.65 %
0.31	[0.22, 0.40]	0.64 %
0.22	[0.14, 0.30]	0.65 %
0.07	[0.05, 0.10]	0.69 %
-0.17	[-0.24, -0.10]	0.66 %
0.72	[0.64, 0.80]	0.65 %
0.64	[0.57, 0.70]	0.67 %
0.31	[0.28, 0.35]	0.68 %
0.11	[0.03, 0.18]	0.66 %
0.14	[0.09, 0.18]	0.68 %

Supplementary file 5. ANOVA analysis

ANOVA of Economic Regions (k'=148)

Factors	Levene test		Variance test		Welch's Variance test		Partial η²
	Levene	*p*	*F*	*p*	*F*	*p*	
Somatization	2.167	0.094	5.261**	0.002	4.646**	0.007	0.098
Obsessive-compulsive	1.057	0.369	5.941**	0.001	5.340**	0.003	0.109
Interpersonal sensitivity	1.425	0.238	3.342*	0.021	3.299*	0.029	0.064
Depression	2.346	0.075	6.142**	0.001	6.204**	0.001	0.112
Anxiety	1.523	0.211	4.519**	0.005	4.313**	0.009	0.085
Hostility	1.410	0.242	2.253	0.085	2.167	0.105	0.044
Phobic anxiety	1.175	0.321	6.770**	0.001	6.331**	0.001	0.122
Paranoid ideation	1.640	0.183	4.785**	0.003	4.848**	0.005	0.090
Psychoticism	0.230	0.875	6.444**	0.001	6.205**	0.001	0.117
Total score	1.401	0.025	6.021**	0.001	5.565	0.002	0.110

Note: k', number of studies for subgroup (including valid mixed samples); Partial η², small effect (0.01), moderate effect (0.06), large effect (0.14)

ANOVA of Urban/Rural Areas (k'=129)

Factors	Levene test		Variance test		Welch's Variance test		Partial η²
	Levene	*p*	*F*	*p*	*F*	*p*	
Somatization	0.362	0.548	0.313	0.577	0.309	0.579	0.003
Obsessive-compulsive	0.678	0.412	5.134*	0.025	5.316*	0.023	0.040
Interpersonal sensitivity	0.698	0.405	5.084*	0.026	5.306*	0.023	0.040
Depression	0.000	0.984	4.924*	0.028	5.039*	0.027	0.038
Anxiety	0.305	0.582	4.736*	0.031	4.621*	0.034	0.037
Hostility	0.103	0.749	1.227	0.270	1.212	0.273	0.010
Phobic anxiety	0.434	0.511	6.381*	0.013	5.845*	0.018	0.049
Paranoid ideation	0.768	0.383	6.608*	0.011	6.814*	0.010	0.051
Psychoticism	0.674	0.413	8.590**	0.004	8.894**	0.003	0.065
Total score	0.027	0.871	6.111*	0.015	6.049*	0.015	0.047

Note: k', number of studies for subgroup (including valid mixed samples); Partial η², small effect (0.01), moderate effect (0.06), large effect (0.14)

Supplementary file 6. Liner regression

Linear regression for SCL-90 factors (1)

Factors	Lag 0 (VIF: 8.655, 2.349, 6.148)				Lag 1 (VIF:13.82, 3.823, 9.648)			
	R^2	*F*	*p*	*DW*	R^2	*F*	*p*	*DW*
Somatization	0.062	0.393	0.759	2.201	0.128	0.878	0.471	2.200
Obsessive-compulsive	0.079	0.517	0.676	2.041	0.095	0.631	0.605	2.044
Interpersonal sensitivity	0.001	0.004	1.000	2.461	0.032	0.201	0.894	2.434
Depression	0.023	0.144	0.932	2.155	0.043	0.268	0.847	2.162
Anxiety	0.013	0.081	0.970	2.181	0.028	0.175	0.912	2.173
Hostility	0.019	0.117	0.949	2.096	0.034	0.211	0.887	2.096
Phobic anxiety	0.003	0.020	0.996	2.251	0.022	0.137	0.937	2.243
Paranoid ideation	0.015	0.088	0.966	2.148	0.036	0.224	0.878	2.155
Psychoticism	0.012	0.073	0.973	2.134	0.048	0.302	0.824	2.135
Total score	0.018	0.113	0.952	2.183	0.045	0.285	0.835	2.186

Note: DW, Durbin-Watson test; VIF, variance inflation factor.

Ridge regression for SCL-90 factors with time lag of 2 years

Factors	Lag 1		
	R^2	*F*	*p*
Somatization	0.122	0.833	0.493
Obsessive-compulsive	0.093	0.617	0.613
Interpersonal sensitivity	0.028	0.170	0.915
Depression	0.043	0.267	0.848
Anxiety	0.027	0.170	0.916
Hostility	0.034	0.210	0.888
Phobic anxiety	0.020	0.120	0.947
Paranoid ideation	0.036	0.233	0.879
Psychoticism	0.046	0.291	0.832
Total score	0.044	0.279	0.840

Linear regression for SCL-90 factors (2)

Factors	Lag 2 (VIF: 6.367, 1.839, 5.025)				Lag 3 (VIF: 6.562, 2.127, 6.365)			
	R^2	*F*	*p*	*DW*	R^2	*F*	*p*	*DW*
Somatization	0.110	0.743	0.540	2.221	0.152	1.078	0.383	2.379
Obsessive-compulsive	0.087	0.575	0.639	2.034	0.095	0.630	0.605	2.062
Interpersonal sensitivity	0.024	0.150	0.928	2.437	0.033	0.202	0.894	2.462
Depression	0.040	0.253	0.858	2.162	0.047	0.297	0.827	2.178
Anxiety	0.028	0.170	0.915	2.172	0.037	0.233	0.872	2.214
Hostility	0.031	0.195	0.899	2.101	0.036	0.222	0.880	2.116
Phobic anxiety	0.018	0.108	0.954	2.257	0.031	0.195	0.899	2.317
Paranoid ideation	0.025	0.157	0.924	2.139	0.036	0.223	0.879	2.146
Psychoticism	0.042	0.266	0.849	2.142	0.053	0.338	0.798	2.189
Total score	0.041	0.258	0.855	2.193	0.055	0.351	0.789	2.232

Note: DW, Durbin-Watson test; VIF, variance inflation factor.
